# Enhanced Properties of 3D-Printed Graphene Oxide Nanocomposites through Itaconic Acid Polyester Grafting

**DOI:** 10.1021/acsapm.5c00014

**Published:** 2025-03-28

**Authors:** Mirko Maturi, Simone Maturi, Alberto Sanz de León, Lorenzo Migliorini, María de la Mata, Tiziana Benelli, Loris Giorgini, Paolo Milani, Mauro Comes Franchini, Sergio Ignacio Molina

**Affiliations:** † Dpto. Ciencia de los Materiales, I. M. y Q. I., IMEYMAT, Facultad de Ciencias, Universidad de Cádiz, 11510 Puerto Real, Cádiz, Spain; ‡ Department of Industrial Chemistry “Toso Montanari”, 9296 University of Bologna, Via P. Gobetti 85, Bologna 40129, Italy; § CIMAINA and Dipartimento di Fisica, Università degli Studi di Milano, Milano 20133, Italy; ∥ Interdepartmental Center for Industrial Research on Advanced Applications in Mechanical Engineering and Materials Technology, CIRI-MAM, University of Bologna, Bologna 40136, Italy

**Keywords:** graphene oxide, nanocomposites, vat photopolymerization, polymer grafting, additive manufacturing

## Abstract

Vat photopolymerization (VP) is a powerful additive manufacturing process to produce high-resolution 3D objects from liquid photocurable resins, but the mechanical performance of its standard materials restricts its use in high-demanding applications. In this study, graphene oxide (GO), a widely investigated nanomaterial, was surface-functionalized by grafting the sustainable and photocurable poly­(butylene itaconate-*co*-adipate) (PBIA) polyester to address these limitations. The covalent grafting of PBIA significantly improved the colloidal stability and dispersibility of GO in photocurable formulations, eliminating the need for extensive homogenization during the formulation of the nanocomposite resin. PBIA-coated GO (GO@PBIA) was easily miscible with VP resins, enabling the fabrication of 3D-printed nanocomposites with superior mechanical properties. At low filler concentrations (0.05 wt %), the GO@PBIA composites increased their elastic modulus up to 57% and tensile strength up to 100% compared to the base polymer, outperforming analogous composites prepared with unmodified GO. Surface modification also enhanced the deformability of the matrix, making these composites suitable for applications under tensile and flexural loads. Optical and morphological analyses confirmed the homogeneous distribution of GO@PBIA within the polymer matrix, demonstrating improved filler–matrix interactions, while electrical conductivity measurements proved that the surface modification approach proposed does not affect the conductive conjugated π system of the nanomaterial. This work highlights the potential of polymer-grafted GO as a multifunctional nanofiller to enhance the mechanical properties and processability of VP-based materials, paving the way for their use in high-performance applications.

## Graphical abstract

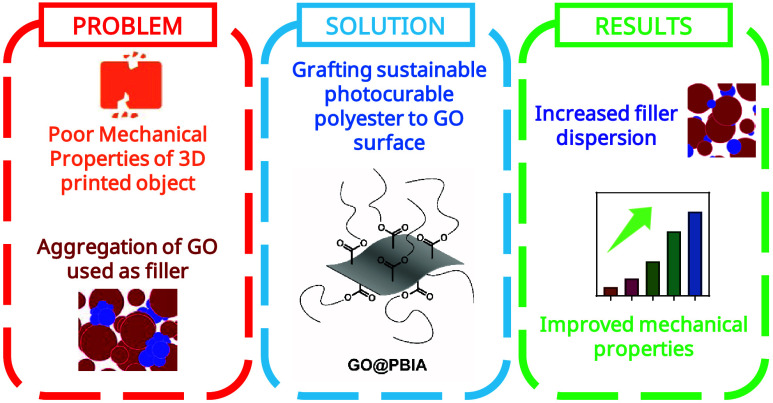

## Introduction

Additive manufacturing, also known as 3D printing or rapid prototyping, is a modern transformative set of technologies that allows for the layer-by-layer fabrication of complex geometries from digital models, eliminating the need for traditional tooling and allowing for customized part design.^1,2^ Among the different 3D printing processes available in the market and the manufacturing industry, vat photopolymerization (VP) technologies have emerged as a leading method due to their ability to produce highly precise parts with fine detail resolution, finding extensive application in industries such as healthcare (dental, prosthetics), aerospace, and consumer electronics due to its precision and material flexibility.^3,4^ In a typical VP 3D printer, a vat is filled with liquid photopolymer resin, and a light source, typically UV or visible, is directed onto the resin in a layer-by-layer fashion to selectively cure specific areas of each layer based on the design of the object to be manufactured. After each layer is cured, the build platform moves incrementally to allow the next layer to be cross-linked. While VP allows one to produce parts with smooth surface finishes, high resolution, and the ability to print a wide range of complex geometries, it faces limitations in terms of the properties of the printed materials. Commercial VP resins have limited mechanical strength, impact resistance, and thermal stability. These material weaknesses restrict the use of VP-printed parts in demanding applications where toughness and durability are essential.^5,6^ This issue is often addressed by including nanoadditives in the currently available photocurable resins, thus leading to 3D-printed nanocomposite materials with improved mechanical performances and, in some cases, new functional properties.^7,8^ Such nanofillers are often inorganic nanostructures such as carbon nanotubes,^9–11^ graphene,^12–14^ graphite,^15^ metal oxides,^16–18^ or silica,^19,20^ but also organic and biopolymeric such as cellulose,^21,22^ chitosan,^23^ or other biomasses. This approach has enabled the development of functional materials that go beyond the limitations of conventional resins. For example, the incorporation of conductive nanoparticles allowed for the fabrication of electrically conductive parts,^24^ while ceramic nanofillers can improve thermal resistance and wear properties, thus expanding the fields of applications of VP to biomedicine (e.g., tissue engineering scaffolds), electronics (e.g., 3D-printed circuit boards), and high-performance mechanical applications (e.g., aerospace components).^25^

Among the different filler materials available, graphene oxide (GO) is attracting the attention of researchers thanks to its significant effects on the mechanical properties of its nanocomposites, even when its content in the polymer matrix is in the part per ten thousand range.^26,27^ Typically produced through the chemical oxidation of graphite, GO bears oxygenated functionalities on its structure, which partially disrupt the electrical conductivity typical of graphene and graphite structures. However, the electrical properties of GO-based nanocomposites can be significantly tuned depending on the processing and modification approaches applied to its structure.^28^ Despite its potential, GO faces significant challenges when it is incorporated into polymer matrices. One of the primary issues is its poor compatibility with hydrophobic polymers due to the hydrophilic nature of its oxygen-containing functional groups. This causes difficulties in achieving uniform dispersion and strong interfacial bonding with the polymer matrix, which ultimately leads to a limited improvement in the properties of the matrix compared to those of the composite. Additionally, GO has a strong tendency to aggregate due to van der Waals interactions and π–π stacking between graphene layers, resulting in nonuniform material properties and reduced mechanical performance in the final composite.^29,30^ To overcome these challenges, surface modification strategies are needed to improve the compatibility of GO with various polymer systems, allowing for the establishment of strong interactions between the polymer network and the reinforcement.

Surface modification of GO is relatively simple, as it bears a variety of reactive functionalities such as hydroxylic, epoxy, and carboxylic groups, which can be promptly reacted with a wide variety of chemical groups. The extensive formation of covalent or H-bonds between the filler’s surface and the matrix network leads to a more efficient matrix-to-filler load transfer when the composite is subjected to mechanical stress, and therefore an overall improvement of its mechanical properties compared to pristine GO, which can only interact with the polymer matrix through weak intermolecular forces.^31,32^ Furthermore, functionalization can be tailored to either preserve or modify GO’s conductivity, depending on the desired application.

In the recent literature, the surface modification of GO to improve its compatibility with VP resins is mainly performed by producing (meth)­acrylic acid-functionalized GO, which can actively participate in the photopolymerization taking place during the 3D printing process.^26,33,34^ Such approaches, which differ in the strategy employed to achieve surface functionalization, led to the obtainment of reinforced composites with improved mechanical properties with low GO contents. However, none of these studies report the effect of surface modification on the electrical properties of the composites as well as the colloidal stability of the GO-loaded liquid mixtures. On the other hand, surface stabilization of GO with dispersing aminated copolymers has allowed for the obtainment of stable GO suspensions in photoresins and of highly conductive composites, but the improvement of the mechanical properties was significantly more limited in the absence of a covalent network connecting the filler and the polymer matrix.^35^

In this work, we present a strategy to grow sustainable poly­(butylene itaconate-*co*-adipate)—PBIA—photocurable polyester chains on the surface of graphene oxide to simultaneously achieve improved colloidal stability and dispersibility of GO in photocurable mixtures and full covalent integration of GO in the photopolymer network. After thorough characterization of the materials, the GO-loaded photocurable mixtures were 3D printed at different loadings in different commercially available resins for VP, in order to evaluate the effect of PBIA grafting onto the GO surface on the mechanical properties of the 3D-printed nanocomposites. Finally, the effect of the surface modification on the electrical conductivity of the nanomaterial was evaluated.

## Materials and Methods

Graphene oxide (GO) was purchased from Graphenea (San Sebastian, Spain). The rest of the chemicals were purchased from Sigma-Aldrich (St. Louis, MO) and used as received. XYZ UV-curable Clear Resin was employed as the rigid resin matrix, while XYZ UV-curable Flexible Resin was employed as the flexible resin matrix.

### Synthesis of Carboxymethylated Graphene Oxide (GO–COOH)

Surface-carboxylated graphene oxide (GO–COOMe) was prepared by the reaction of the surface oxygenated moieties with chloroacetic acid in alkaline environment, as previously reported.^36^ In a typical procedure, 5 g of GO were dispersed in 800 mL of water by extensive sonication in an ultrasound bath (1 h), followed by the sequential addition of a first solution of 50 g of NaOH in 100 mL of water and a second solution of 50 g of chloroacetic acid in 100 mL of water. Carboxymethylation reaction was allowed to take place by stirring at room temperature for 2 h. Then, the mixture was acidified with concentrated HCl until pH 1 to induce the agglomeration of protonated GO–COOH, which was then separated by centrifugation and thoroughly washed with water (3x 10 min at 5000 rpm). The precipitate was then washed once with acetone to remove most of the moisture and dried for 6 h at 50 °C. Obtained mass: 4.95 g (yield = 99%).

### Synthesis of Carboxymethylated Graphene Oxide Methyl Ester (GO–COOMe)

GO–COOH from the previous step was subjected to Fisher esterification to produce the corresponding methyl ester. Typically, 4.95 g of GO–COOH were dispersed in 100 mL of methanol by extensive sonication in an ultrasound bath (1 h), followed by the addition of 10 mL of concentrated H_2_SO_4_ (98 wt %). The mixture was then refluxed for 1 h under stirring conditions and inert atmosphere. Once cooled to room temperature, the acid was neutralized by the addition of a saturated NaHCO_3_ solution until pH 8. Finally, the obtained carboxymethylated graphene oxide methyl ester (GO–COOMe) was separated by centrifugation and repeated washings with water (3 × 10 min at 5000 rpm). The precipitate was then washed once with acetone to remove most of the moisture and dried for 6 h at 50 °C. Obtained mass = 4.55 g (yield = 92%)

### Synthesis of Free and GO Surface-Grafted Poly­(butylene itaconate-*co*-adipate) (PBIA and GO@PBIA)

In a three-necked 1-L round-bottomed flask, 4.5 g of GO–COOMe were dispersed in 1,4-butanediol (56.8 g, 0.63 mol) with magnetic stirring and sonication (30 min). Then, dimethyl itaconate (79.0 g, 0.5 mol), dimethyl adipate (21.8 g, 0.125 mol), and dibutyl tin­(IV) oxide (DBTO, 1.57 g, 6.3 mmol) were sequentially added. The mixture was heated to 175 °C under Ar atmosphere for 1 h, and then, vacuum (10 mmHg) was applied for another 1.5 h. During the reaction time, the flask was attached to a distillation condenser which allowed for the removal of the produced MeOH. Once cooled, the reaction mixture appeared as a black viscous liquid, which was precipitated in a 1:1 mixture of MeOH and iPrOH. The obtained polymer was then dissolved in the minimum amount of dichloromethane and reprecipitated, twice. Finally, the GO-loaded polyester GO@PBIA was dried under a high vacuum and stored at +4 °C. Obtained mass = 70.6 g. Yield = 57%, calculated as the ratio between the mass of GO@PBIA and the difference between the total mass of monomers and GO–COOMe and the amount of methanol distilled during the synthesis (38.4 g). Free PBIA was prepared analogously but in the absence of GO–COOMe. Obtained mass = 68.7 g. Yield = 58% calculated as before, considering that the amount of MeOH distilled during the polymerization was equal to 39.1 g in this case.

### Quantification of GO Content in GO@PBIA

A sample of GO@PBIA (2.21 g) was treated with 10 mL of boiling 2 M NaOH for 15 min. After it was cooled, the suspension was repeatedly centrifuged and washed with water until neutrality of the supernatant was reached. Then, the pellet was dried in an oven at 80 °C until a constant weight was achieved. The GO content was determined as the ratio between the mass of GO and the mass of GO@PBIA. As control samples, the same treatment was performed on pristine GO and free PBIA. In the first case, no mass loss was detected due to alkaline treatment, while in the second case, no precipitate was detected, thus confirming the reliability of the employed quantification method.

### Preparation of Photocurable Formulations and VAT Photopolymerization 3D Printing

Photocurable formulations were prepared by mixing GO@PBIA or pristine GO with free PBIA and commercial photocurable resins, varying the GO content from 0.01 to 0.5%. Formulated nanocomposite mixtures were denoted with an alphanumeric code reporting the letter M or U referring to the use of modified or unmodified GO, respectively, the letter R or F referring to the use of rigid or flexible resin as the matrix, and a number corresponding to the concentration of GO expressed as permyriad. A summary of the composition of the various formulations is provided in Table S1. In the case of GO@PBIA, the mixture was homogenized through simple manual stirring, while when pristine GO was used as the additive, the mixture has been processed by high-speed homogenization (Ultra-Turrax, 5 krpm, 10 min) followed by extensive sonication in a USC500T Ultrasonic Cleaner for 30 min. Then, the mixture was poured into the vat of the VP printer (Phrozen Sonic Mini 8K) and different objects were manufactured by using a 405 nm LCD-LED screen with a XY resolution of 22 μm and a layer height of 50 μm. Computer-assisted design (CAD) files of 1BA tensile testing specimens according to ISO 527, bending testing specimens according to ISO 178 and complex structures were loaded using the Chitubox v1.9.5 software. The 3D-models were converted into a GCODE file suitable for 3D printing. The printed objects were then separated from the printer platform and washed with an isopropyl alcohol/acetone 1:1 mixture. Postprocessing of the samples was carried out for 60 min in a chamber equipped with a 405 nm light source and a power of 1.25 mW/cm^2^ (FormCure, Formlabs), previously heated to 60 °C.

### Physical, Chemical, and Mechanical Characterization

^1^H-, ^13^C-, ^1^H–^1^H-COSY, and ^1^H–^13^C-HSQC NMR spectra were obtained at 298 K on a Bruker (14.01 T, 600.13 MHz) NMR spectrometer. In all recorded spectra, chemical shifts are reported in parts per million of frequency relative to the residual solvent signals for both nuclei (^1^H: 7.26 ppm and ^13^C: 77.16 ppm for CDCl_3_). ^13^C NMR analysis was performed using the ^1^H broadband decoupling mode. ATR-FTIR analyses were performed with a PerkinElmer Spectrum Two spectrophotometer, equipped with a Universal ATR accessory; all spectra were recorded as an average of 20 scans (range 4000–400 cm^–1^ with a resolution of 0.5 cm^–1^). ζ-potential measurements were conducted in DTS1060C-Clear disposable zeta cells at 25 °C on a Malvern Zetasizer-Nano-S. Data are reported as mean ± SD of three independent measurements. Rotational viscosity measurements were performed on an Anton Paar MCR102 Rheometer with a cone–plate CP50-1 configuration (1° angle and 50 mm diameter). Shear stress and viscosity as functions of the shear rate were measured at 25 °C. Viscosity was measured as a function of temperature with a constant rotational frequency of 1 Hz in the temperature range +3/+40 °C and a heating rate of 5 °C/min. Size exclusion chromatography (SEC)/gel permeation chromatography (GPC) was performed on a HPLC Lab Flow 2000 apparatus, equipped with an injector Rheodyne 7725i, a Phenomenex Phenogel 5μ MXL column, and a Shodex R1-71 refractive index detector. HPLC grade tetrahydrofuran (THF) was used as the eluent with a flow rate of 1 mL/min. The system was calibrated with polystyrene (PS) standards covering a molar mass range from 300 to 30000 g/mol (Merck). The mechanical properties of the printed composites were measured in a Shimadzu AGS-X universal testing machine. Tensile and flexural testing of the specimens were carried out at 1 mm/min, in agreement with ISO 527 and ISO 178, respectively. For flexural testing, the measurement was stopped at 20% deformation if the material did not break, since at high deformation, the sample starts slipping on its supports, making the force reading less representative of the structural properties of the material. SEM measurements were carried out on Au-coated specimens (5 nm coating thickness) in a Thermo Fisher Scientific NOVA NanoSEM 450 microscope equipped with a field-emission gun. TEM measurements, including scanning transmission electron microscopy (STEM) imaging and electron energy loss spectroscopy (EELS), were carried out on a Thermo Fisher Scientific TALOS F200X instrument provided with a Schottky-type field-emission gun (X-FEG) and a Gatan Continuum energy filter (suitable for the EELS mapping), operated at 200 kV. The colloidal suspensions containing either GO or GO@PBIA were directly drop-casted onto the TEM carbon membranes. Electrical conductivity measurements were conducted on the samples UF50 and MF50, four specimens each, printed as 1 mm thick discs. IV curves have been acquired using gold electrodes (contact area of 0.81 cm^2^), a DC generator (DC power supply 3371E, LKB) to provide an applied potential up to 1 kV and a digital multimeter (34410A, Agilent) to measure the flowing current. For each IV curve, linear regression has been employed to calculate the corresponding conductivity (s) value of each specimen. Conductivity values (and their corresponding errors) for the UF50 and MF50 formulations have been calculated using the formula for the weighted average. Thermogravimetric analysis (TGA) was performed on a Q50 (TA Instruments, New Castle, DE) thermoscale. Following a typical procedure, a temperature sweep of up to 600 °C was performed by using a constant rate of 10 °C/min. All of the TGA experiments were carried out under a constant nitrogen flow.

## Results and Discussion

### Synthesis and Characterization of GO-Grafted and Free PBIA

The surface of GO is notoriously rich in oxygenated organic functional groups such as carboxylic acid, epoxide, and hydroxy moieties.^37^ However, such inhomogeneity of the chemical nature of the GO surface prevents the development of extensive surface modification strategies without a prior alteration through surface chemistry. For this purpose, commercial GO was functionalized with carboxymethyl residues upon treatment with an alkaline solution of sodium chloroacetate, which reacted with surface hydroxy groups to produce the corresponding glycolic acid ether.^36^ Furthermore, epoxides are easily cleaved in strongly alkaline environments into the corresponding vicinal diols, thus offering additional hydroxy groups able to be carboxymethylated.^38^ Then, the abundant carboxylic residues were esterified with methanol, hence preparing the surface moieties for polymer grafting (Figure 1a). The effectiveness of GO chemical modification steps was assessed by FTIR spectroscopy (Figure 1b), and the main absorption bands have been assigned based on recent literature data.^39^ At first, by analyzing the spectral region where the C–O stretching modes resonate (900–1300 cm^–1^), the presence epoxides in pristine GO (950 cm^–1^) is clearly observed, along with their expected disappearance after carboxymethylation. Furthermore, while the peak for C–O ether stretching is present in all samples (at 1050 cm^–1^), an additional peak at 1230 cm^–1^ appears only in GO–COOMe. This is related to the ester C–O stretching, thus confirming the effectiveness of the esterification of the GO surface. On the other hand, by looking at the region between 1500 and 1800 cm^–1^, it is possible to track the presence and nature of C=C and C=O unsaturations. The aromatic C=C bond stretching peak is identified at 1610 cm^–1^ and is present in all samples, while the C=O stretching band at 1720 cm^–1^ appears in all samples. The evolution of the relative intensities of the two peaks can be explained by considering that while GO contains both C=C and C=O bonds, its carboxylation in alkaline environment leads to the formation of surface carboxylate groups (COO^–^), which resonate at frequencies comparable to those of aromatic C=C moieties.^40^ After esterification, the C=O vibration frequency moves to higher wavenumbers, leading to the increased intensity of the band at 1720 cm^–1^ and the expenses of the peak at 1610 cm^–1^ in the GO–COOMe sample. The effectiveness of carboxymethylation is further confirmed by the appearance of aliphatic CH_2_ stretching peaks between 2900 and 3000 cm^–1^. Additionally, the significant reduction of the broad O–H absorption band in GO–COOMe provides further evidence for the success of the esterification step.

**Figure 1:**
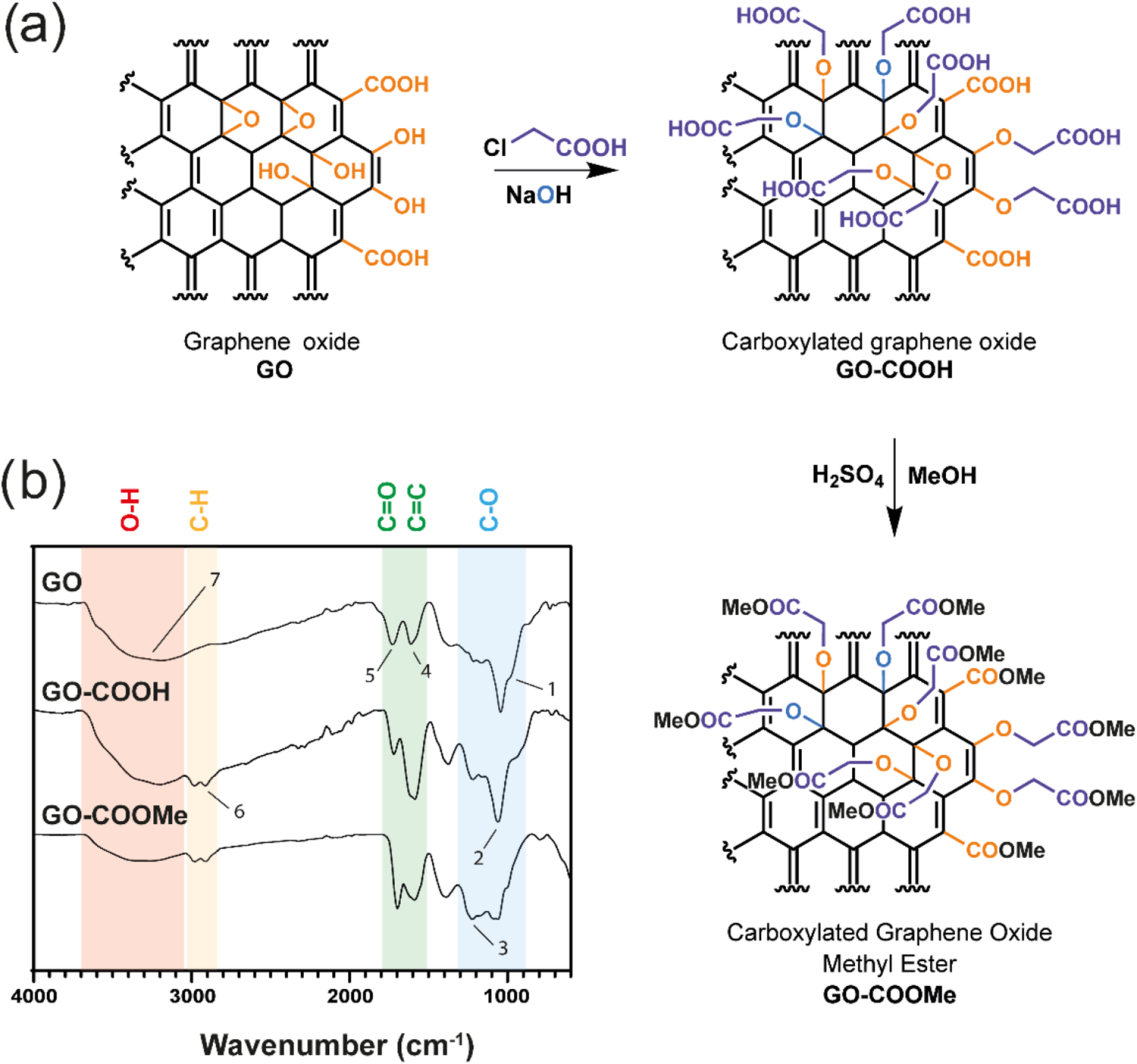
Synthesis of carboxymethylated graphene oxide methyl ester. (a) Schematics of the surface modification strategies implemented on the GO surface. (b) ATR-FTIR analysis of GO, GO–COOH, and GO–COOMe. Labeled absorption bands correspond to wavenumbers: (1) 950 cm^–1^, (2) 1050 cm^–1^, (3) 1230 cm^–1^, (4) 1610 cm^–1^, (5) 1720 cm^–1^, (6) 2912 and 2980 cm^–1^, and (7) 3250 cm^–1^.

Furthermore, the expected results were further supported by ζ-potential measurements. In fact, while pristine GO showed a negative ζ-potential of −17.7 ± 1.2 mV due to the oxygenated surface moieties, this value increased to −27.7 ± 1.8 mV for GO–COOH, as can be expected for the replacement of electronegative OH groups with the net charges of partially dissociated carboxylic acid groups. Similarly, the esterification of such groups with methanol eliminated most net charges from the surface, and therefore, the ζ-potential dropped to −9.11 ± 0.80 mV.

The morphology and composition of GO and GO–COOMe were further addressed by means of scanning transmission electron microscopy (STEM) (Figures 2 and S1). Whereas the commercial GO sheets tend to fold and winkle, leading to micrometer-sized GO bunches (Figure 2a), GO–COOMe (Figure 2b) shows uniform dispersion on the supporting carbon membrane (TEM grid), meaning that these sheets remain flatter. The chemical analyses performed by EELS allow depicting the distribution of the species within the sample. Consequently, GO–COOMe sheets can be unambiguously distinguished from the carbon supporting membrane by tracking the oxygen signal (cyan maps in Figure 2). Notably, as well as providing the chemical composition of the material at subnanometer scale, EEL spectra contain valuable information on the atomic bonding and coordination state. Thus, the analyzed materials show differentiated EELS shapes at both the C and the O K-edges, related to the presence of different functional groups. Many types of C bonds within the material (i.e., C–C, C=C, C–O, C=O, C–H, vacancy environment, etc.) render enriched spectra whose overall shape will also depend on their actual relative amount.^41^ Therefore, the accurate analysis of the EELS C K-edge in these materials becomes challenging, particularly in inhomogeneous samples. It must be considered that chemical inhomogeneities within single sheets will provide differentiated EEL signatures, as it is shown for the raw GO when comparing the C signal from different locations (see bottom panels of Figure 2). In contrast, GO–COOMe sheets show a more homogeneous composition, likely due to the efficient functionalization. The O K-edge, showing up at about 530 eV, also evidence fingerprints from each material, with differentiated contributions of the features at 531, 533, and 538 eV. While the O K-edge spectral shape from raw GO points toward the presence of C–OH bonds, GO–COOMe mainly resembles C–O–C reported spectra, in good agreement with the functionalization performed.^42^

**Figure 2:**
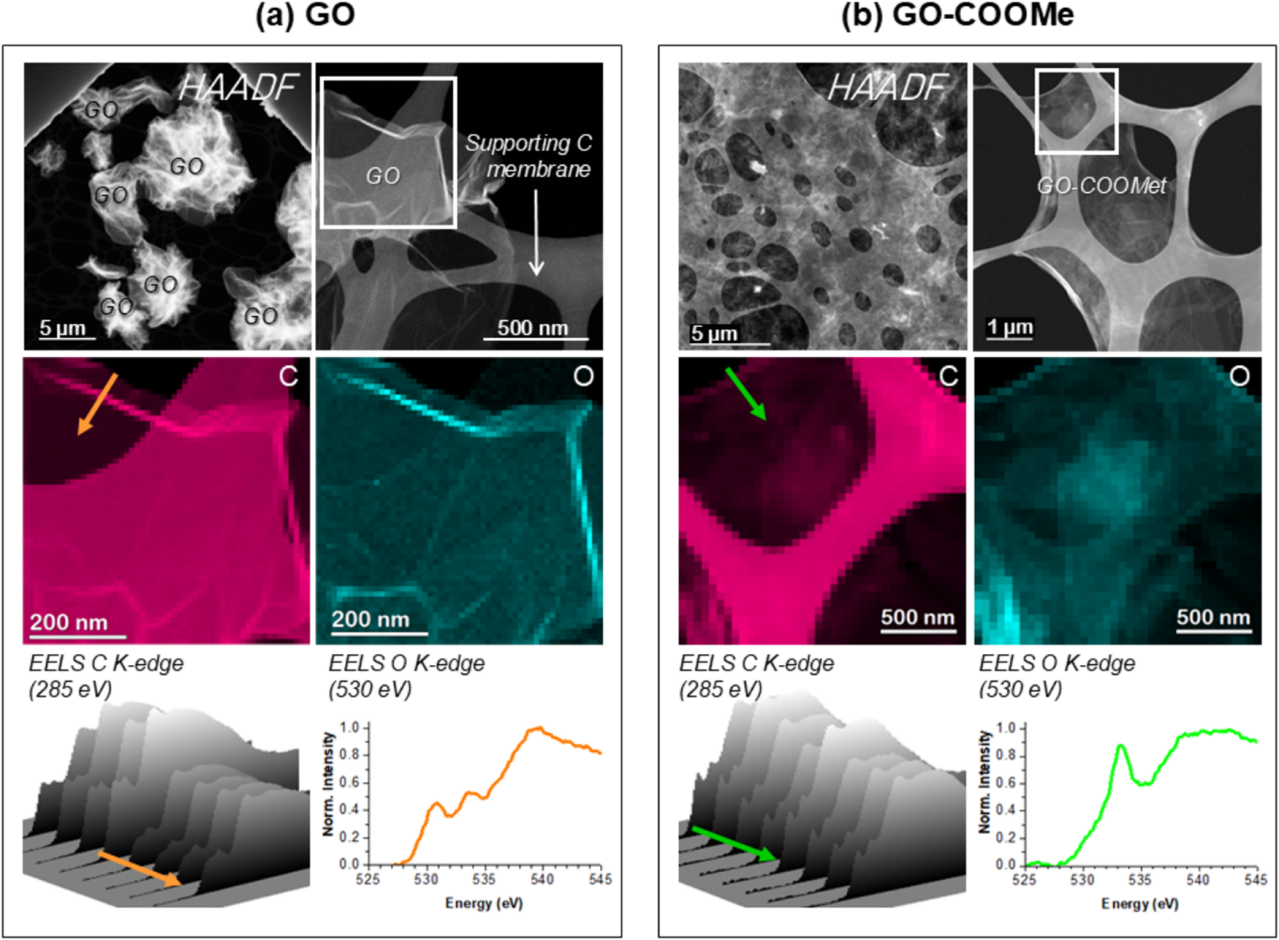
STEM-EELS analyses of GO (a) and GO–COOMe (b) samples, showing their morphology (top) and chemical constituent distribution (middle), along with representative EELS spectra from both materials (bottom).

In order to improve the compatibility of GO with the acrylic resins usually employed for VP processes, poly­(butylene itaconate-*co*-adipate), PBIA, was grafted from the surface of GO–COOMe by exploiting the reactivity of its surface methyl esters (Figure 3). PBIA is a photocurable biobased copolyester of itaconic and adipic acid with 1,4-butanediol, synthesized following a previously reported tin-catalyzed polytransesterification approach where dimethyl esters of itaconic acid and other aliphatic diacids react with diols to produce the corresponding polyester, with methanol as the condensation product.^43,44^ Adipic acid was included to reduce the total itaconic acid content and therefore to improve the mechanical performances of the final photocured resins, based on previously reported studies.^43,44^ When the polymerization of itaconic and adipic acid with 1,4-BDO was performed in the presence of GO–COOMe, the surface methyl ester groups competed with the methyl esters of the diacids, thus grafting the linear polyester to the surface of graphene oxide (GO@PBIA).

**Figure 3:**
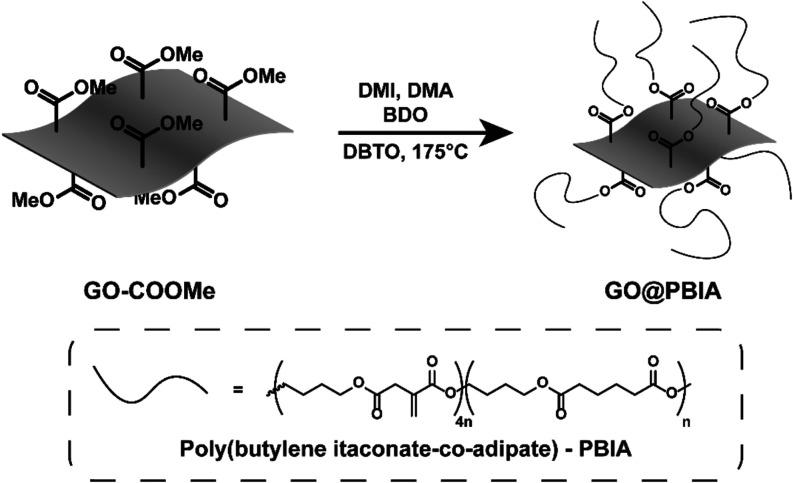
Schematic representation of the grafting of PBIA from the surface of carboxymethylated GO. DMI = dimethyl itaconate, DMA = dimethyl adipate, BDO = 1,4-butanediol and DBTO = dibutyl tin­(IV) oxide.

The polymerization yields (57 and 58% for GO@PBIA and free PBIA, respectively) are in line with what previously reported for the tin-catalyzed polytransesterification of dimethyl itaconate, and it was not affected significantly by the presence of GO–COOMe.^43,44^ The effectiveness of the covalent binding of PBIA macromolecules to the surface of GO was assessed by evaluating the solvent affinity of GO–COOMe vs GO@PBIA. GO–COOMe is easily dispersed in methanol by gentle mixing, but such dispersion was not obtained with GO@PBIA even after extensive stirring and sonication, due to the poor solubility of polyesters in polar alcohols such as methanol (Figure S2). Free PBIA was prepared similarly but in the absence of GO–COOMe, leading to the obtainment of a pale yellow thick liquid. GO content in GO@PBIA was evaluated by depolymerizing surface-grafted PBIA by means of a concentrated alkaline solution at high temperatures, which led to the full saponification of PBIA into its monomers and the obtainment of hydrophilic GO–COOH as a fine suspension. Such analysis revealed that the total GO content of GO@PBIA was equal to 6.23 wt %. Furthermore, no appreciable loss of GO was detected during the purification of the polymerization product, and the GO content measured gravimetrically after alkaline hydrolysis is fully consistent with this hypothesis.

GO@PBIA and PBIA were further characterized by NMR spectroscopy (Figures S3–S5). The analysis revealed the expected chemical structure, and the 4:1 molar ratio between itaconic and adipic monomer units was confirmed by the integration of representative NMR peaks. No significant differences were detected when the NMR spectra of PBIA and GO@PBIA, apart from the relative area underneath the peaks related to terminal groups. Within the intrinsic errors of NMR peak integration, PBIA displayed terminal monomers that are roughly twice as abundant as GO@PBIA. This is consistent with the hypothesis that the presence of GO–COOMe in the reaction mixture did not affect the molecular weight of the polymer chains, since PBIA grafted on the GO surface will display terminal methyl or alcoholic CH_2_ residues only on one side of each macromolecule. This hypothesis holds in the case that no free PBIA was formed during the polycondensation performed in the presence of GO or, if it was formed, it was efficiently removed by purification.

The molecular weight of PBIA was measured by GPC-SEC, revealing a broad molecular weights distribution with $\overset{®}{\mathrm{Mn}}$ = 9300 g/mol and $\overset{®}{\mathrm{Mw}}$ = 19000 g/mol, and therefore a polydispersity index of around 2.0. By cross-evaluation of these values with the obtained GO content of 6.23 wt % in GO@PBIA, it is possible to approximately calculate, based on previously reported literature data,^45,46^ that the described construct bears on average roughly one PBIA chain every 14 ± 5 carbon atoms, corresponding to an effective functionalization and grafting of 7.5 ± 2.5% of the GO surface atoms. This result is fully consistent with the GO surface structure and surface functionalization, suggesting the coherence of the presented results. Details of the calculations are available in the Supporting Information.

Furthermore, the rheology of the prepared polymers was evaluated by employing rotational viscosity measurements (Figure S6). Both PBIA and GO@PBIA were characterized by fully Newtonian behavior up to around 200 s^–1^ where free PBIA started displaying shear-thinning behavior, while this effect is much more limited in the case of GO@PBIA. Furthermore, the latter displays generally lower viscosity compared with free polyester. Both findings can be expected in the case of surface-grafted polymer chains due to several effects occurring at the molecular scale, including conformational restrictions, reduced interchain entanglement, and nanoparticle-induced dilution which cause decreased polymer viscosity when this is grafted on the surface on nano- or microstructures.^47,48^ At high shear rates, interchain entanglement is strongly reduced in free PBIA, and the two systems display practically the same rheological behavior. Furthermore, viscosity decreases significantly when the temperature increases for both PBIA and GO@PBIA.

Finally, the thermal stability of the prepared materials was evaluated by using TGA (Figure S7). By analyzing the thermograms it is evident that the grafting onto GO surface does not alter the thermal stability of PBIA which decomposes in the 300–400 °C temperature range, consistently with what was previously reported for other itaconic acid polyesters.^43^ Furthermore, the thermal decomposition of GO@PBIA generates carbonaceous residues equal to 17.4% of the initial weight, while the residue of free PBIA is only 11.3% of its initial mass. Such a difference (6.1 wt %) is coherent with the quantified GO content in GO@PBIA (6.23%), thus confirming the previously reported data.

### Formulation and 3D Printing of GO-Loaded Photocurable Resins

The prepared and characterized GO@PBIA was formulated by simple mixing with commercial rigid and flexible resins for stereolithography, in order to evaluate its influence on mechanical, optical, and electrical properties of these materials after 3D printing. The concentration of PBIA in the different samples was kept constant at 15 wt % by adding free PBIA in the required amounts, and control samples were prepared by mixing unmodified GO with the corresponding amount of free PBIA and photocurable resins (Table S1). This allowed us to directly correlate variations in mechanical, optical, and electrical properties to the nature of the GO surface and its concentration. It is worth mentioning that while GO@PBIA was a thick paste-like material fully miscible with photocurable resins in all proportions by simple manual mixing, the dispersion of solid GO nanopowder in the photoresins required extensive sonication, high-energy vortexing, and homogenization, until no aggregates were visible to the naked eye. Rheological analysis suggested that the addition of 15 wt % PBIA to commercial resin leads to photocurable mixtures possessing optimal viscosity for applications in VP, since they display fully Newtonian behavior and viscosity below 10 Pa s at temperatures above 15 °C (Figure S8).

The prepared formulations were then 3D printed by using a desktop LCD-LED commercial VP printer. The presence of black, light-absorbing graphene oxide in the photocurable mixtures required an increase in the exposure time per layer to efficiently achieve solid objects with high resolution as the nanofiller absorbed most of the radiation emitted by the LCD light source. Therefore, an optimal irradiation time of 60 s per layer was required to achieve the full polymerization of the liquid photoresin. On the other hand, the presence of GO within the tested concentration range did not significantly impact the printing resolution. However, its surface chemistry demonstrated a notable effect on this aspect (Figure S9). In fact, while the M set of resins allowed for the 3D printing of fine details with very high resolution and detail, this was not the case for U resins, loaded with pristine GO, which failed in successfully printing small features such as hanging bridges, sharp spikes, and thin walls. This is probably due to the poorer colloidal stability of GO compared to GO@PBIA, which causes aggregation and sedimentation of the nanofiller which act as scattering centers dispersing the UV light on a broader area, thus leading to nonoptimal spatial confinement of the photopolymerization front and nonuniform dispersion of the nanofiller in the printed material. For example, the sedimentation of GO in the printer vat during printing was already evident after 3 h of printing using UR25, while it was not detectable when resin MR25 was used (Figure S10). The lower colloidal stability of GO compared to GO@PBIA not only reflects on poorer printing resolution but also alters the amount of nanomaterial that is effectively incorporated into the polymer composite. In fact, it is possible to assess simply by naked eye that composites with the same nanofiller loadings printed with GO@PBIA appear darker than composites printed with pristine GO (Figure 4). This was more quantitatively assessed by measuring the optical transmittance of 3D-printed samples throughout the visible spectrum (Figure S11). Transmittance measurement revealed a higher transparency of all samples of the U series compared to the M series with equivalent GO concentration, suggesting that the sedimentation of unmodified GO reduces the extent of GO incorporation into the final 3D printed material. This difference was not observed in the uncured resins tested right after thorough mixing (Figure S12), discarding the possibility that the observed differences in the 3D printed materials are caused by an underestimation of GO content in GO@PBIA.

**Figure 4:**
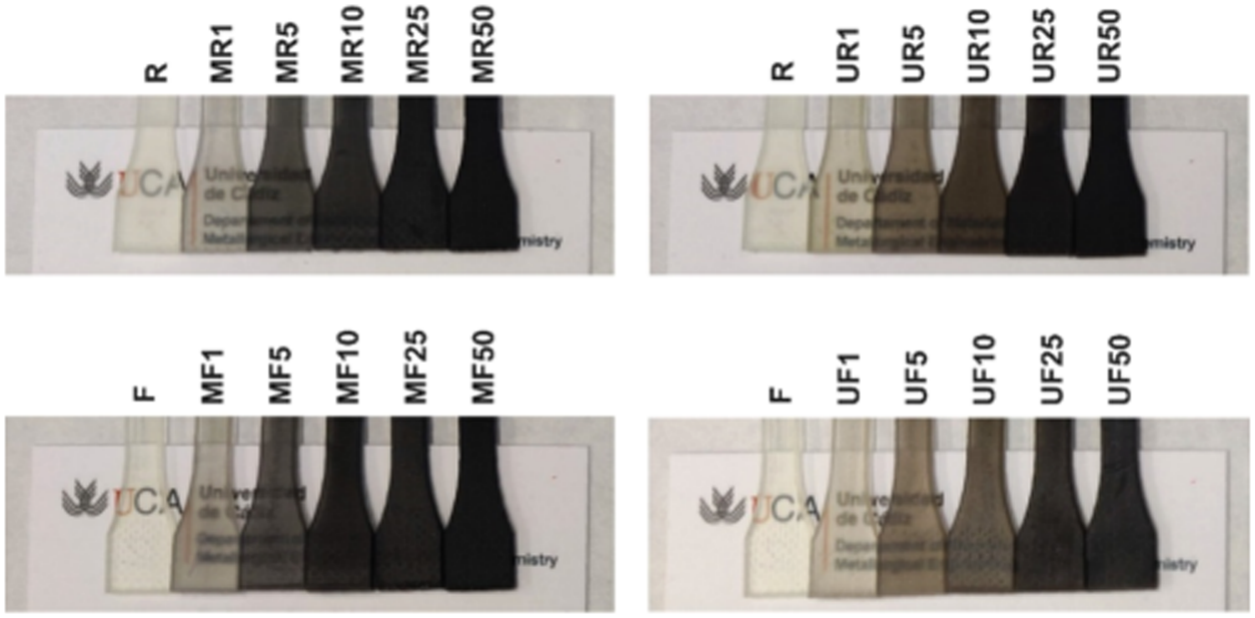
Optical camera photographs of 3D-printed tensile test specimens showing the optical transparency of the composites.

Electrical conductivity measurements provided additional evidence of enhanced colloidal stability of the GO following polymer grafting. In fact, for the flexible composites with the highest GO content, UF50 and MF50, electrical conductivity measurements (Table S2) revealed that while neither reached the percolation threshold and both remained electrically insulating at 0.5 wt %, MF50 exhibited an electrical conductivity approximately 3 times greater than UF50. This finding suggests that the polymer grafting strategy preserved the electrically conductive conjugated π-system of GO.

Thermogravimetric analysis (TGA) was performed on 3D printed samples with 0 and 0.5 wt % of either GO or GO@PBIA in either flexible or rigid resin to evaluate the influence of the presence of the nanofiller and its surface chemistry on the thermal stability of the 3D printed materials (Figure S13). For the thermograms, it is possible to observe that, while unmodified GO leads to a small but detectable reduction in the thermal stability of the 3D printed resin, anticipating its thermal degradation of about 10 °C, GO@PBIA does not affect the thermal stability of the polymer matrix. This effect is probably related to the catalytic properties of bare GO, which has been reported to be able to accelerate the degradation of polymer matrices such as PLA.^49^

Finally, 3D printed specimens underwent tensile and flexural testing to assess the influence of GO addition and surface modification on the mechanical properties of the nanocomposites (Figure S14). The evolution of mechanical properties such as elastic modulus, elongation at break, tensile strength, and tensile toughness with increasing GO or GO@PBIA content is plotted in Figure 5 and collected in Tables S3 and S4. It is worth pointing out that deformation at break and flexural toughness of flexible resins was not reported, as the materials did not break up to 20% deformation. Calculating flexural toughness in such cases is not practical, as toughness is conventionally defined as the energy absorbed until failure. Without a clear failure point, energy measurement becomes arbitrary rather than an intrinsic material property, leading to misleading comparisons.

**Figure 5:**
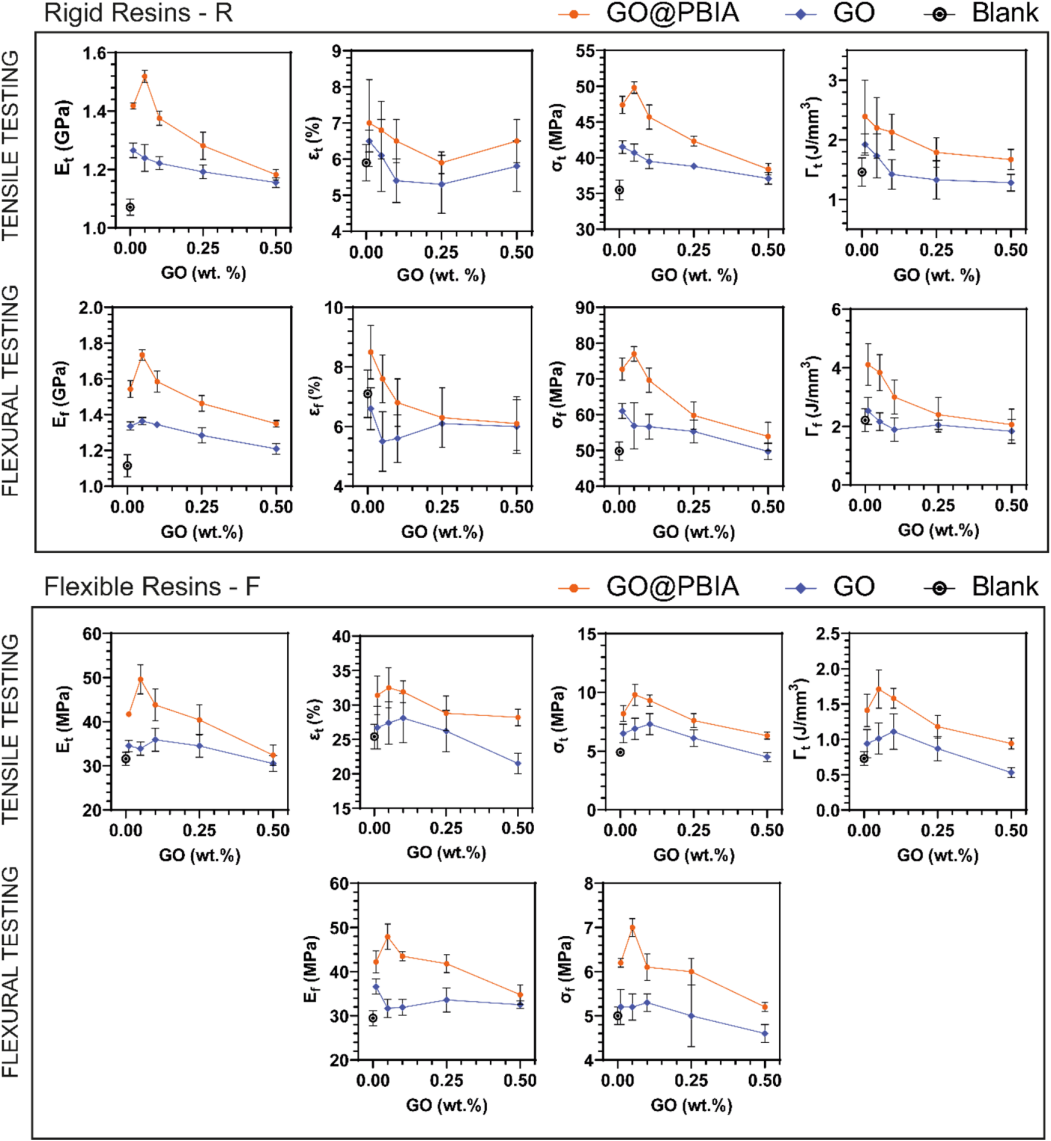
Tensile and flexural testing of GO- and GO@PBIA-loaded formulations in rigid (top) and flexible (bottom) resins. The blank always corresponds to materials achieved by 3D printing resin R or F. Error bars represent the standard deviation of each data set (*n* = 5). *E*_t_ = tensile modulus, ε_t_ = elongation at break, σ_t_ = tensile strength, Γ_t_ = tensile toughness, *E*_f_ = flexural modulus, ε_f_ = deformation at break, σ_f_ = flexural strength, and Γ_f_ = flexural toughness.

By comparing the performances of GO@PBIA-loaded nanocomposites with the ones prepared using pristine GO, it is clear that the surface modification of GO with itaconic acid-based polyesters significantly enhances all mechanical properties compared to pristine GO, demonstrating superior reinforcement across a range of concentrations. This effect is particularly pronounced at a low GO concentration of 0.05 wt % in the rigid matrix, where the stiffness, strength, and toughness achieved their highest values, increasing by up to 42, 40, and 51% under tensile testing and by 56, 55, and 74% under flexural testing, respectively. The higher improvement of properties compared to tensile properties can be related to the combination of tensile and compressive stresses in bending, which allows for progressive damage and energy absorption, delaying failure. In contrast, tensile testing leads to a more uniform stress distribution, causing a more abrupt and brittle failure.^50^ The remarkable enhancement at 0.05 wt % suggests that optimal filler–matrix interactions are achieved at this concentration, facilitated by the covalent bonding of PBIA chains to the GO surface and their integration into the cross-linked photopolymer network. Pristine GO, although it improves stiffness (e.g., Young’s modulus increases by 8–18%), shows limited effects on ductility and toughness, as weak intermolecular forces dominate its interaction with the matrix. In contrast, GO@PBIA facilitates superior load transfer during mechanical stress, as the grafted polyester chains actively bridge the matrix and filler, mitigating stress concentrations and enhancing deformability. Notably, this improvement is observed across both rigid and flexible resin matrices, while to different extents. As flexible photoresins are characterized by a lower degree of cross-linking, which allows for the photopolymer chains to rearrange themselves in space upon the application of external mechanical stresses, they are not able to efficiently transfer such mechanical stress to the nanofiller, thus limiting its contribution to the overall material properties. On the other hand, the surface modification of GO with long photocurable polyester chains allows for a more intimate covalent connection between the photopolymer network and the nanomaterial, thus leading to an improved matrix-to-filler load transfer. Furthermore, in both resin types, the superior mechanical performance of GO@PBIA-loaded nanocomposites diminishes at higher filler concentrations (>0.1 wt %), likely due to the stabilization of free radicals on GO’s conjugated π systems, as previously reported.^51^ At high filler loadings, GO nanocomposites often become more brittle due to reduced matrix ductility, poor interfacial adhesion, and stress concentration effects. Excess fillers restrict plastic deformation, while weak interfaces and agglomerates create defects that promote crack initiation. As a result, toughness decreases due to the material’s lower ability to absorb energy before failure.^52^

When compared to different GO surface modification strategies, the grafting of PBIA has been demonstrated to positively affect all mechanical properties to a greater extent, and some previous studies achieved more limited improvements with lower GO contents. For example, Ramirez-Soria et al. demonstrated improvements of tensile properties using GO modified with nonsustainable aminated silanes in photocurable resins for VP 3D printing at concentrations as low as 0.01 wt %, but the incorporation of the nanofiller did not affect significantly elastic modulus and tensile strength of the composites.^53^ On the other hand, a comparison with VP nanocomposites based on different acrylated nanostructures reveals that the observed enhancement of mechanical properties is more pronounced than most of the previously reported examples.^54–56^

The surface fracture of the flexible- and rigid-based composites after tensile tests were analyzed by SEM, containing either GO or GO@PBIA fillers (Figures 6, S15, and S16). The surface morphologies of both resins show plain differences. The lower magnification SEM images evidence the substantial deformation of the material at the fracture surfaces for the rigid resin-based composites compared to the flexible ones. Whereas flexible resin suffers from strong elastic deformation resulting in smooth fracture surfaces, the rigid specimens show scarps (sharp steps at the boundary between two adjacent crack planes^57^) and river lines as a consequence of their brittle fracture. We can distinguish two differentiated behaviors of the GO at both resins when they are analyzed at higher magnifications. On the one hand, the GO is able to slide within the flexible matrix until some extent during the tensile test creating the observed grooves at the fracture surfaces, in good agreement with the likely rearrangement of the polymer chains. On the other hand, the GO sheets remained anchored at the matrix within rigid resins, likely hindering the crack propagation more efficiently. Interestingly, the roughness of the fracture surface of rigid nanocomposites increases with the addition of GO, which correlates with the embrittlement of the material. Both grooves and cracks at the flexible and rigid resins, respectively, increase with GO content. Therefore, the GO sheets embedded in the resins may assist material deformation upon tensile strength, contributing to enhancing their mechanical properties at low loadings.

**Figure 6:**
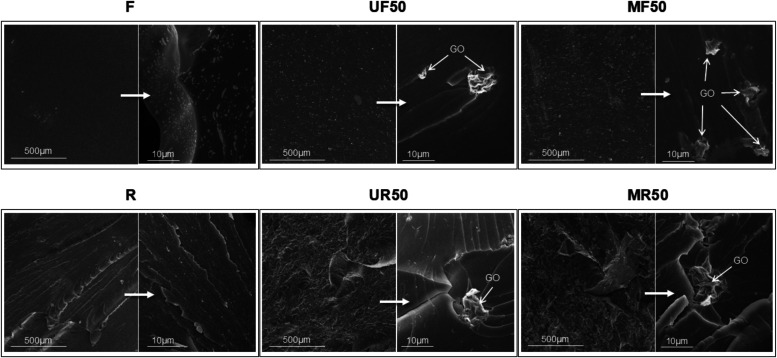
SEM images of the tensile fracture surface of specimen printed with blank resins (F, R), with 0.5% unmodified GO (UF50 and UR50), and with 0.5% GO as GO@PBIA (MF50 and MR50).

## Conclusions

In this study, we developed a novel surface modification strategy for graphene oxide (GO) by grafting a sustainable and photocurable poly­(butylene itaconate-*co*-adipate) (PBIA) polyester, enhancing its compatibility with vat photopolymerization (VP) resins. This modification significantly improved GO’s dispersibility, eliminating the need for extensive homogenization and enabled the formulation of stable photocurable nanocomposite resins. The successful incorporation of PBIA-grafted GO (GO@PBIA) into VP resins led to the fabrication of 3D-printed nanocomposites with outstanding mechanical enhancements. At just 0.05 wt % loading, the GO@PBIA nanocomposites exhibited up to a 42% increase in elastic modulus and a 40% improvement in tensile strength compared to the base polymer, outperforming composites with unmodified GO. The covalent grafting of PBIA to GO facilitated superior matrix–filler interactions, resulting in uniform dispersion and enhanced deformability under mechanical stress. Furthermore, electrical conductivity measurements confirmed that the surface modification did not disrupt GO’s conjugated π-system, preserving its potential for multifunctional applications. This work demonstrates that PBIA-grafted GO effectively overcomes key challenges in incorporating GO into VP resins, offering a scalable strategy to enhance the mechanical performance and processability of 3D-printed nanocomposites. These findings pave the way for the use of polymer-functionalized GO in high-performance applications, broadening the scope of vat photopolymerization in structural and functional material design.

## Supplementary Material



## Notes

Detailed composition of prepared formulations, NMR spectra, rheological analysis, TGA, optical camera pictures of 3D-printed samples, visible light transparency spectra, and tensile and flexural stress–strain curves and data (PDF)
