# Supplementary material for: Enhanced Properties of 3D-Printed Graphene Oxide Nanocomposites through Itaconic Acid Polyester Grafting
Source: ACS Appl Polym Mater. 2025 Mar 28;7(7):4371–82. doi: 10.1021/acsapm.5c00014 (PMC12150230; doi:10.1021/acsapm.5c00014)
Supplement: Supplementary file 1 [file ap5c00014_si_001.pdf]

SUPPORTING INFORMATION

Enhanced Properties of 3D-Printed Graphene Oxide Nanocomposites through Itaconic Acid Polyester Grafting

Mirko Maturi[1,], Simone Maturi[2], Alberto Sanz de León[1], Lorenzo Migliorini[3], María de la Mata[1], Tiziana Benelli[2,4], Loris Giorgini[2,4], Paolo Milani[3], Mauro Comes Franchini[2], and Sergio Ignacio Molina[1]*

[1] Dpto. Ciencia de los Materiales, I. M. y Q. I., IMEYMAT, Facultad de Ciencias, Universidad de Cádiz, 11510 Puerto Real, Cádiz, Spain.

[2] Department of Industrial Chemistry "Toso Montanari", University of Bologna, Via P. Gobetti 85, 40129, Bologna, Italy

[3] CIMAINA and Dipartimento di Fisica, Università degli Studi di Milano, Milano 20133, Italy.

[4] Interdepartmental Center for Industrial Research on Advanced Applications in Mechanical Engineering and Materials Technology, CIRI-MAM, University of Bologna, Bologna 40136, Italy.

Corresponding author details

Mirko Maturi, PhD. Email: mirko.maturi@uca.es

Sample	Resin	Filler	Filler content (wt.%)	GO content (wt.%)	GO-bound PBIA content (wt.%)	Added free PBIA content (wt.%)	Total PBIA content (wt.%)
**R**	Rigid	-	-	-	-	15	15
**MR50**	Rigid	GO@PBIA	7.94	**0.5**	7.44	7.56	15
**MR25**			3.97	**0.25**	3.72	11.28	15
**MR10**			1.59	**0.1**	1.49	13.51	15
**MR5**			0.794	**0.05**	0.744	14.26	15
**MR1**			0.159	**0.01**	0.149	14.85	15
**UR50**	Rigid	GO	0.5	**0.5**	-	15	15
**UR25**			0.25	**0.25**	-	15	15
**UR10**			0.1	**0.1**	-	15	15
**UR5**			0.05	**0.05**	-	15	15
**UR1**			0.01	**0.01**	-	15	15
**MF50**	Flexible	GO@PBIA	7.94	**0.5**	7.44	7.56	15
**MF25**			3.97	**0.25**	3.72	11.28	15
**MF10**			1.59	**0.1**	1.49	13.51	15
**MF5**			0.794	**0.05**	0.744	14.26	15
**MF1**			0.159	**0.01**	0.149	14.85	15
**UF50**	Flexible	GO	0.5	**0.5**	-	15	15
**UF25**			0.25	**0.25**	-	15	15
**UF10**			0.1	**0.1**	**-**	15	15
**UF5**			0.05	**0.05**	-	15	15
**UF1**			0.01	**0.01**	-	15	15
**F**	Flexible	-		-	-	15	15

Table S1. Composition of the prepared GO-loaded photocurable formulations

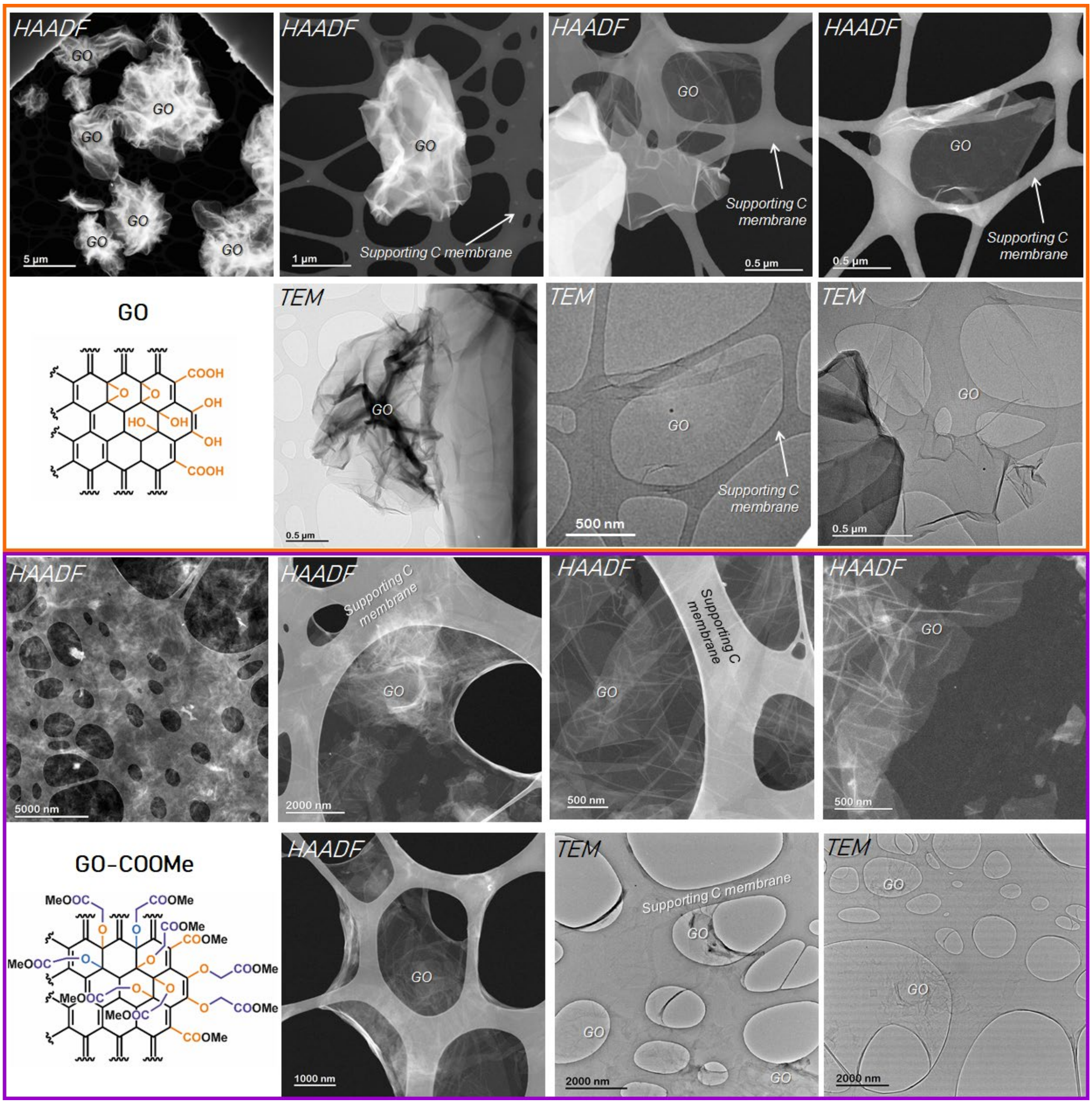

Figure S1. (S)TEM images of GO (top panel) and GO-COOMe (bottom panel), taken at low magnifications in order to show the differentiated morphology of the sheets.

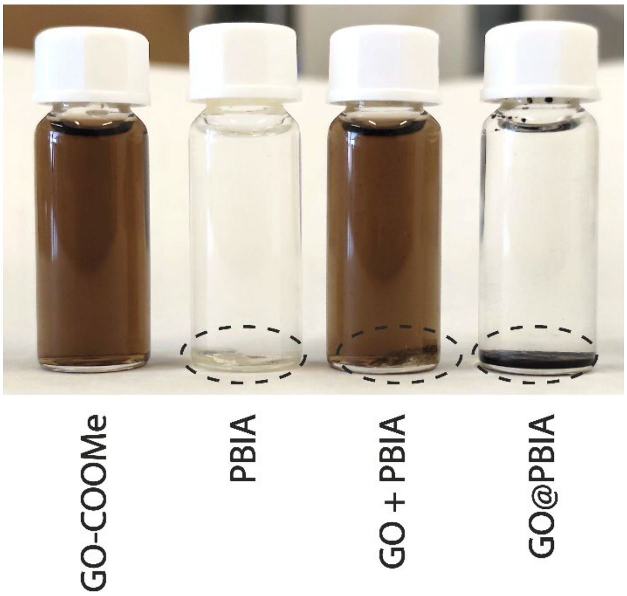

Figure S2. Solvent affinity of GO-COOMe, PBIA, GO@PBIA, and a mixture of GO-COOMe and PBIA in methanol.

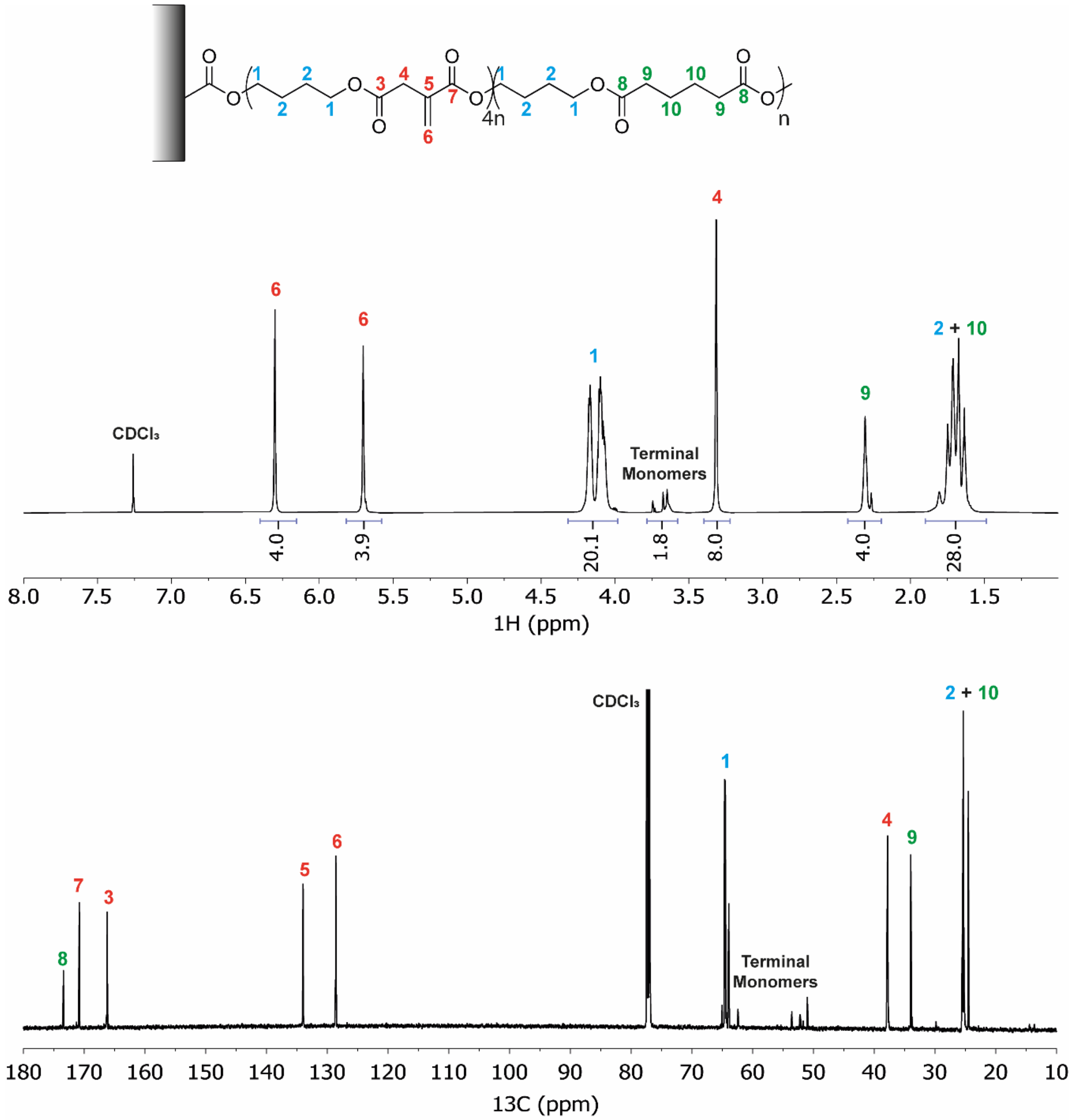

Figure S3. ^{1}H- (600 MHz, $CDCl_3$) and ^{13}C- (150 MHz, $CDCl_3$) NMR spectra of GO@PBIA, with the corresponding spectral assignments.

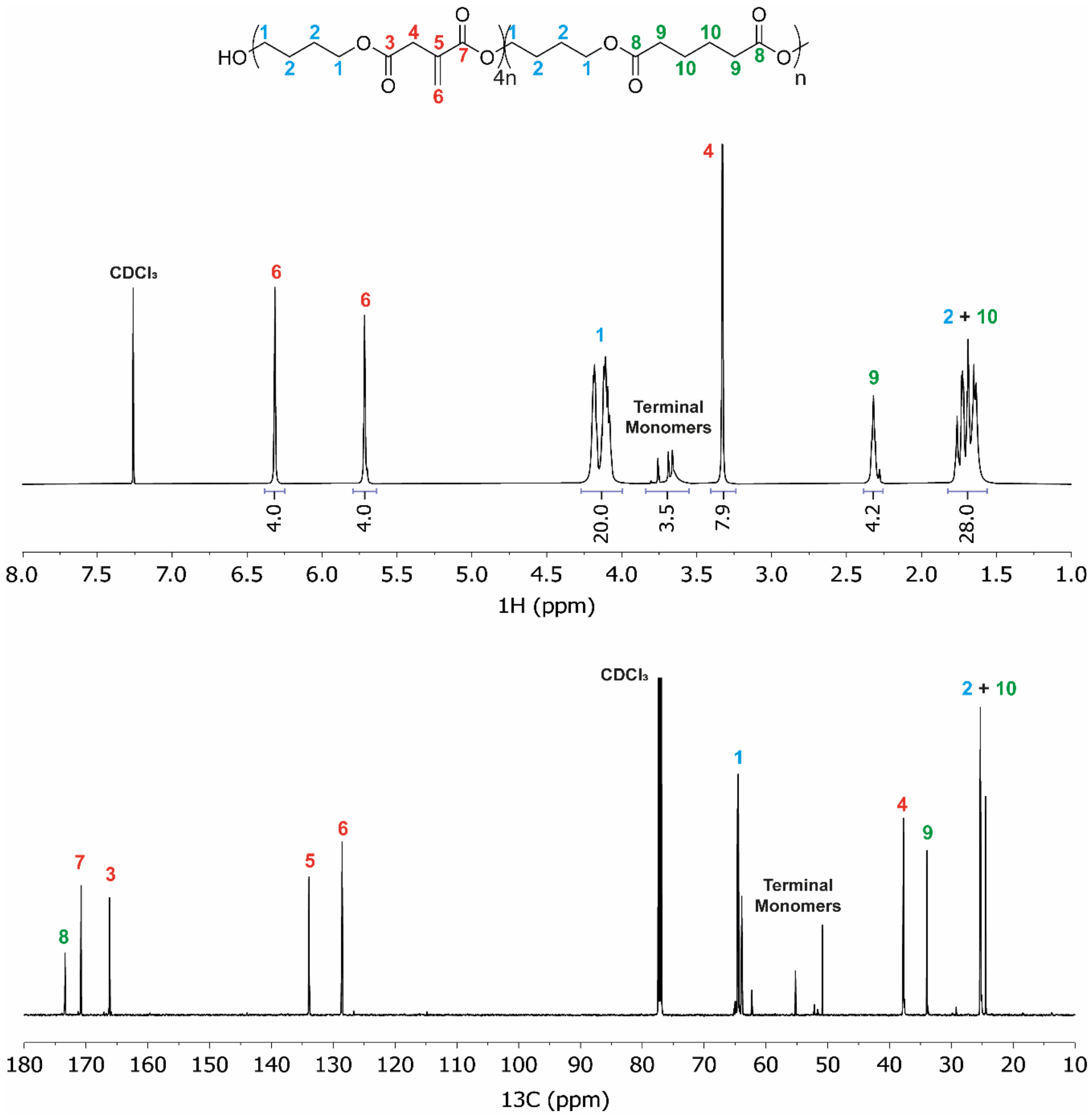

Figure S4. ^{1}H- (600 MHz, $CDCl_3$) and ^{13}C- (150 MHz, $CDCl_3$) NMR spectra of PBIA, with the corresponding spectral assignments.

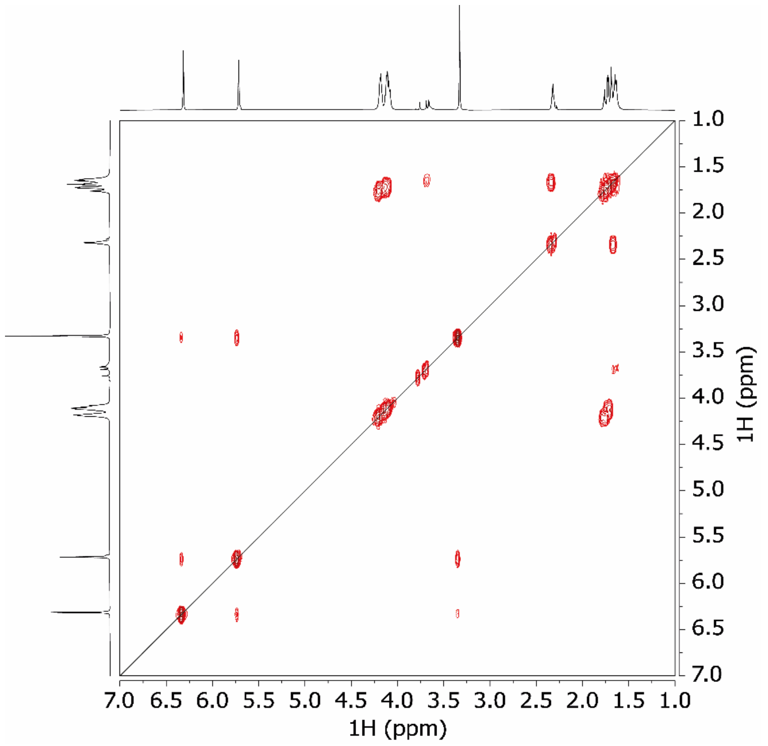

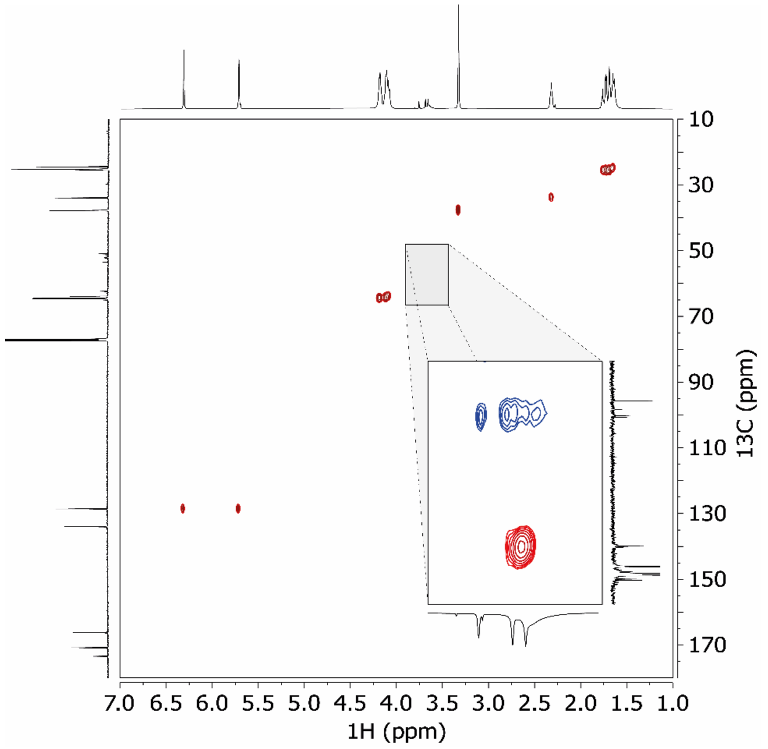

Figure S5. ^{1}H-^{1}H COSY (600 MHz, $CDCl_3$, top) and ^{1}H-^{13}C HSQC (600 MHz, $CDCl_3$, bottom) NMR analysis of GO@PBIA. Positive peaks are colored in red and are related to CH and CH_3 groups, while negative peaks are colored in blue and are related to CH_2 groups. HSQC spectrum displays an expansion of the region assigned to the terminal monomers, which allows to discriminate between the peaks related to terminal methyl ester and the ones related to terminal alcoholic CH_2 moieties.

CALCULATION OF THE SURFACE DISTRIBUTION OF PBIA ON GO@PBIA

The detected amount of GO in GO@PBIA resulted as equal to $6.23\,wt.\%$. This means that 100 g of GO@PBIA contain $6.23\,g$ of GO and $93.77\,g$ of PBIA. The specific surface area (SSA) of GO depends on several factors, including surface defects and degree of exfoliation, but using as a reference various literature data[1,2], a value of $SSA = 200\,m^2/g$ represents a good approximation. Using this range of values, we can calculate that $6.23\,g$ bear a total surface area of

$$A = 6.23\,g \cdot SSA \approx 1250\,m^2$$ **Equation S1**

On this surface area, we grafted 93.77 g of PBIA, with a molecular weight (MW) ranging from 9300 to 19000 g/mol, as determined by GPC-SEC. This corresponds to a number n of macromolecules:

$$n = \frac{93.77}{MW} \cdot 6.022 \cdot 10^{23} = (4.5 \pm 1.5) \cdot 10^{21}\,macromolecules$$ **Equation S2**

Therefore, we can calculate n_A, the number of macromolecules grafted on a unit area of GO:

$$\boldsymbol{n_A} = \frac{n}{A} = (3.6\ \pm 1.2) \cdot 10^{18}\,\frac{macromolecules}{m^2} = \boldsymbol{(3.6 \pm 1.2)\,\frac{macromolecules}{nm^2}}$$ **Equation S3**

Parallelly, the number of carbon atoms per unit area of graphene oxide can be calculated from the hexagonal lattice structure and the C-C bond length in graphene. It should be kept into account that this is an approximate approach, and the real carbon density on graphene oxide is lower than this due to the presence of oxygenated functionalities and structural defects introduced during oxidation. However, for the purpose of this calculation, we can acceptably consider the geometry of pure graphene. The area of the hexagonal unit cell in graphene can be calculated as:

$$A_{hex-cell} = \frac{3\sqrt{3}}{2}a^2 = 0.0525\,nm^2$$ **Equation S4**

Where a is the C-C bond length in graphene ($0.142\,nm$). Since the unit cell of graphene contains 2 atoms, the carbon atom density on the surface of graphene is equal to:

$$\boldsymbol{Surface\ density = \frac{2}{A_{hex-cell}} = 38\,atoms/nm^2}$$ **Equation S5**

And therefore, by combining the obtained results we can approximately determine that GO@PBIA displays one polymer chain every 14 ± 5 carbon atoms. This corresponds to an effective functionalization and grafting of 7.5 ± 2.5 % of the GO surface atoms.

(1) Albers, P. W.; Leich, V.; Ramirez-Cuesta, A. J.; Cheng, Y.; Hönig, J.; Parker, S. F. The Characterisation of Commercial 2D Carbons: Graphene, Graphene Oxide and Reduced Graphene Oxide. *Mater. Adv.* **2022**, *3* (6), 2810–2826. https://doi.org/10.1039/D1MA01023A.

(2) Achawi, S.; Feneon, B.; Pourchez, J.; Forest, V. Structure–Activity Relationship of Graphene-Based Materials: Impact of the Surface Chemistry, Surface Specific Area and Lateral Size on Their In Vitro Toxicity. *Nanomaterials* **2021**, *11* (11), 2963. https://doi.org/10.3390/nano11112963.

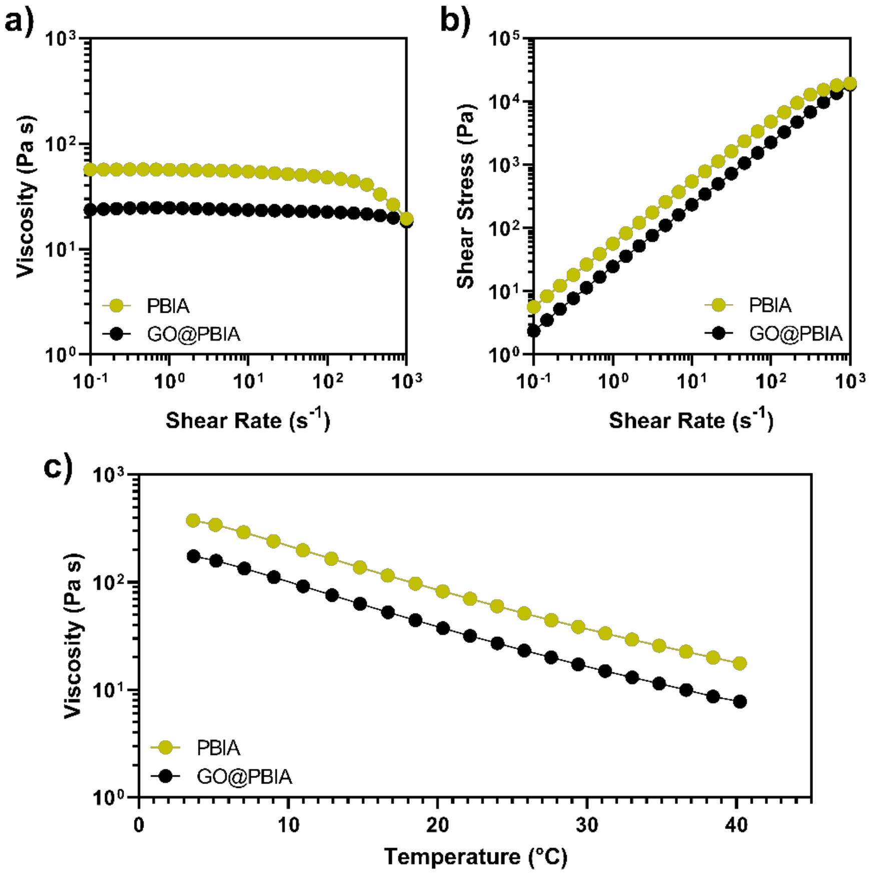

Figure S6. Rheological analysis of GO and GO@PBIA. Viscosity and shear stress as a function of shear rate (a and b) were evaluated at 25°C. Viscosity as a function of temperature (c) was assessed using a constant shear rate of 1 s^{-1}.

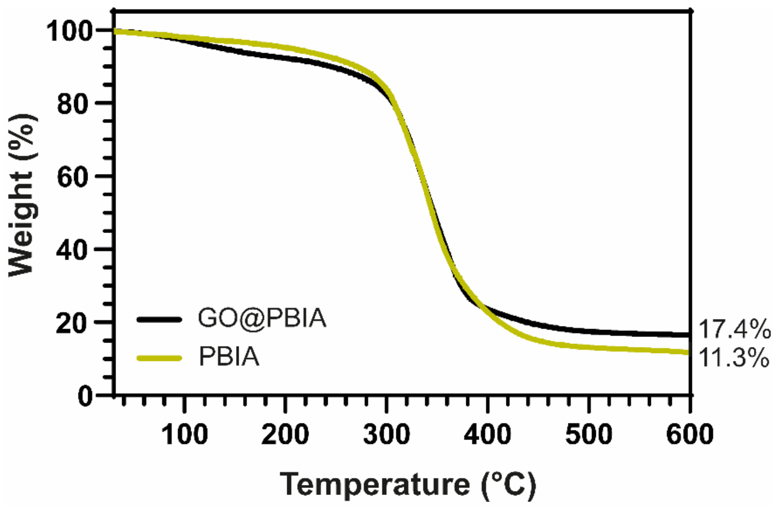

Figure S7. Thermogravimetric analysis of GO and GO@PBIA in N_2 atmosphere. The reported percentages correspond to the residual mass of carbonized material with respect to the initial mass of the sample.

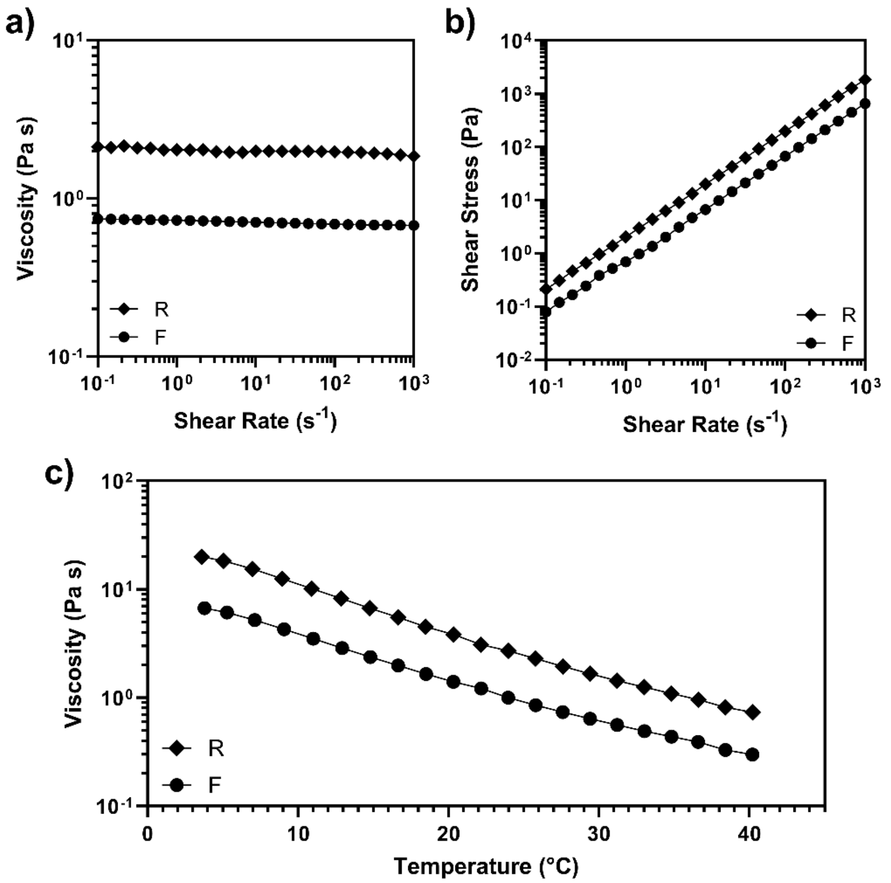

Figure S8. Rheological analysis of resins R and F. Viscosity and shear stress as a function of shear rate (a and b) were evaluated at 25°C. Viscosity as a function of temperature (c) was assessed using a constant shear rate of 1 s^{-1}.

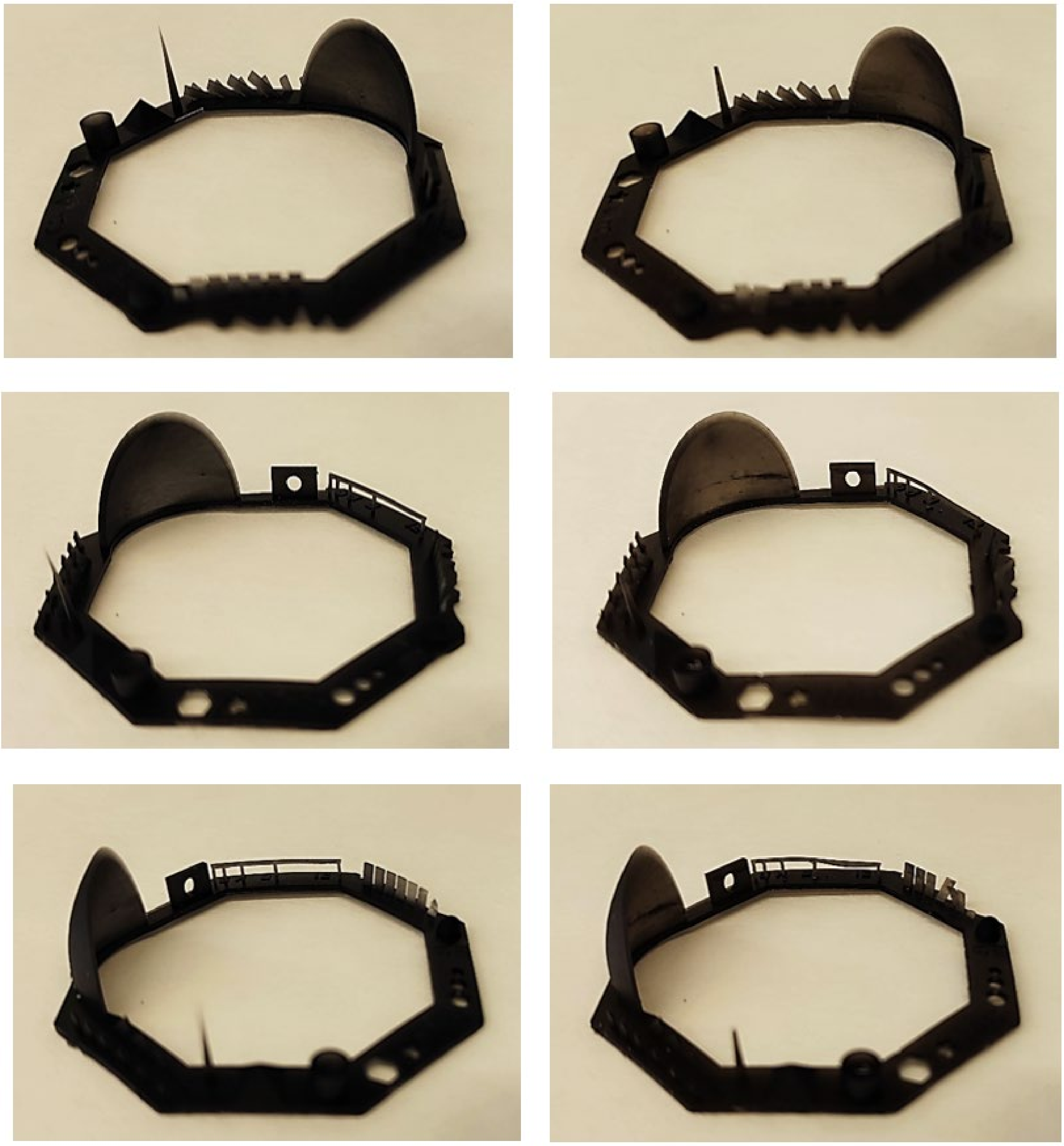

Figure S9 Comparison between the printing performances of resin MR25 (left) vs. UR25 (right). Test print *.stl file was downloaded free of charge from Makerworld.com[1]

[1] (https://makerworld.com/en/models/54359#profileId-56081)

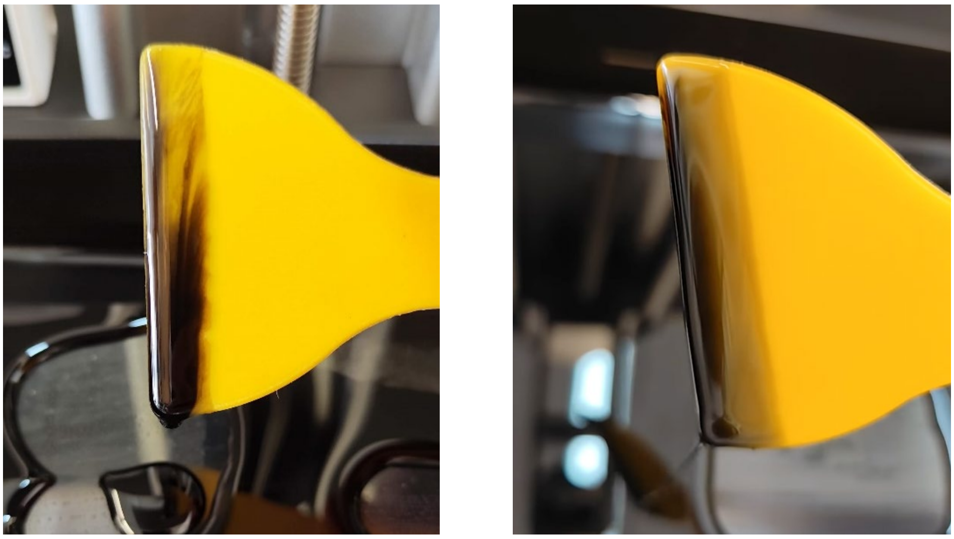

Figure S10. Appearance of the GO-loaded resins UR25 (left) and MR25 (right) collected from the bottom of the resin vat after 3 hours of printing.

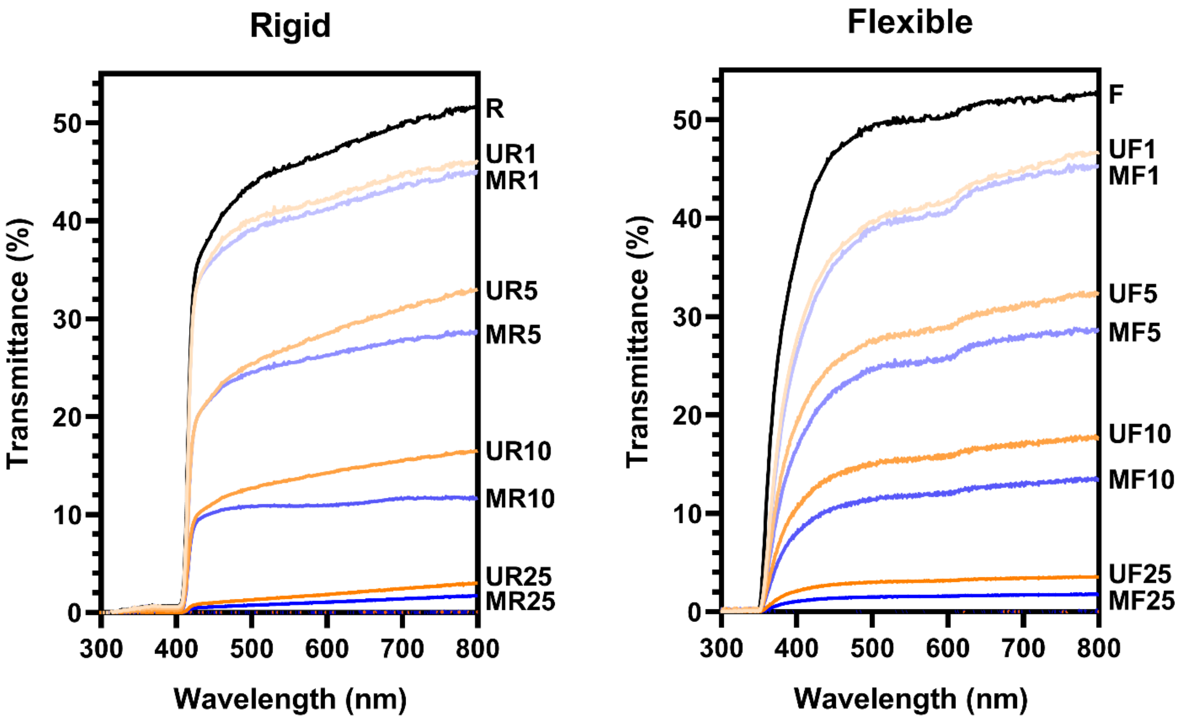

Figure S11. Visible light transparency of the 3D printed specimens. Sample thickness is 2 mm in all cases.

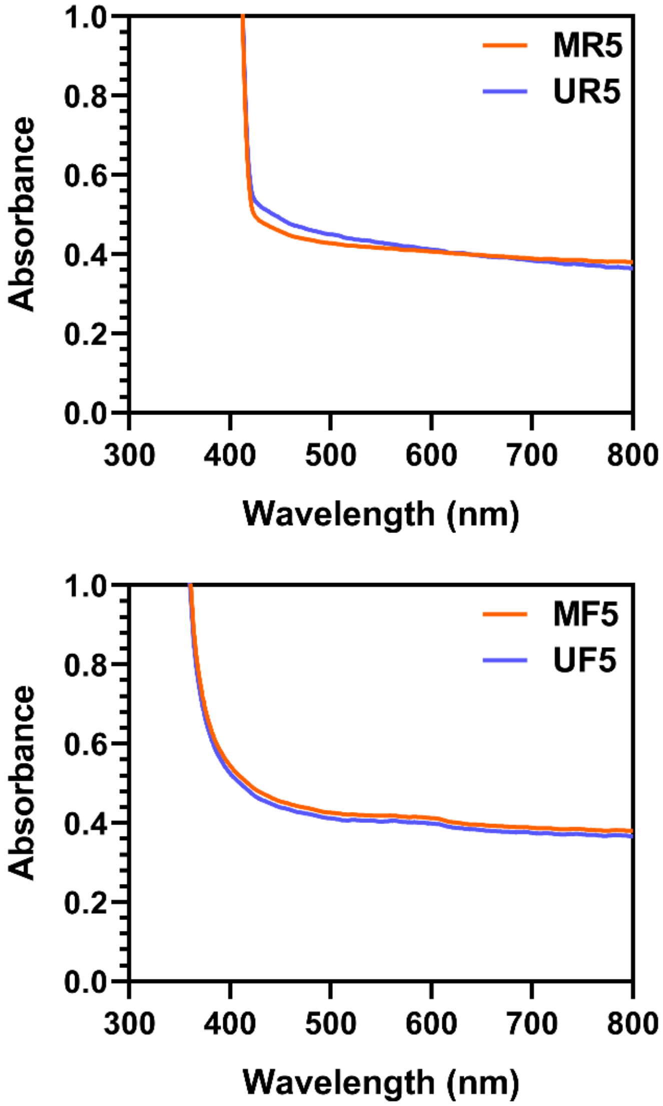

Figure S12. Comparison of the UV-VIS extinction behavior of liquid resins MR5 vs UR5 (top) and MF5 vs UF5 (bottom) diluted 10x in THF (optical path length = 1 cm).

Sample	Electrical conductivity (S/cm)
F	$(8.70 \pm 0.61) \times 10^{-12}$
UF50	$(8.00 \pm 0.10) \times 10^{-11}$
MF50	$(2.36 \pm 0.03) \times 10^{-10}$

Table S2. Measured electrical conductivity of 3D printed specimens. Data are expressed as mean ± SD.

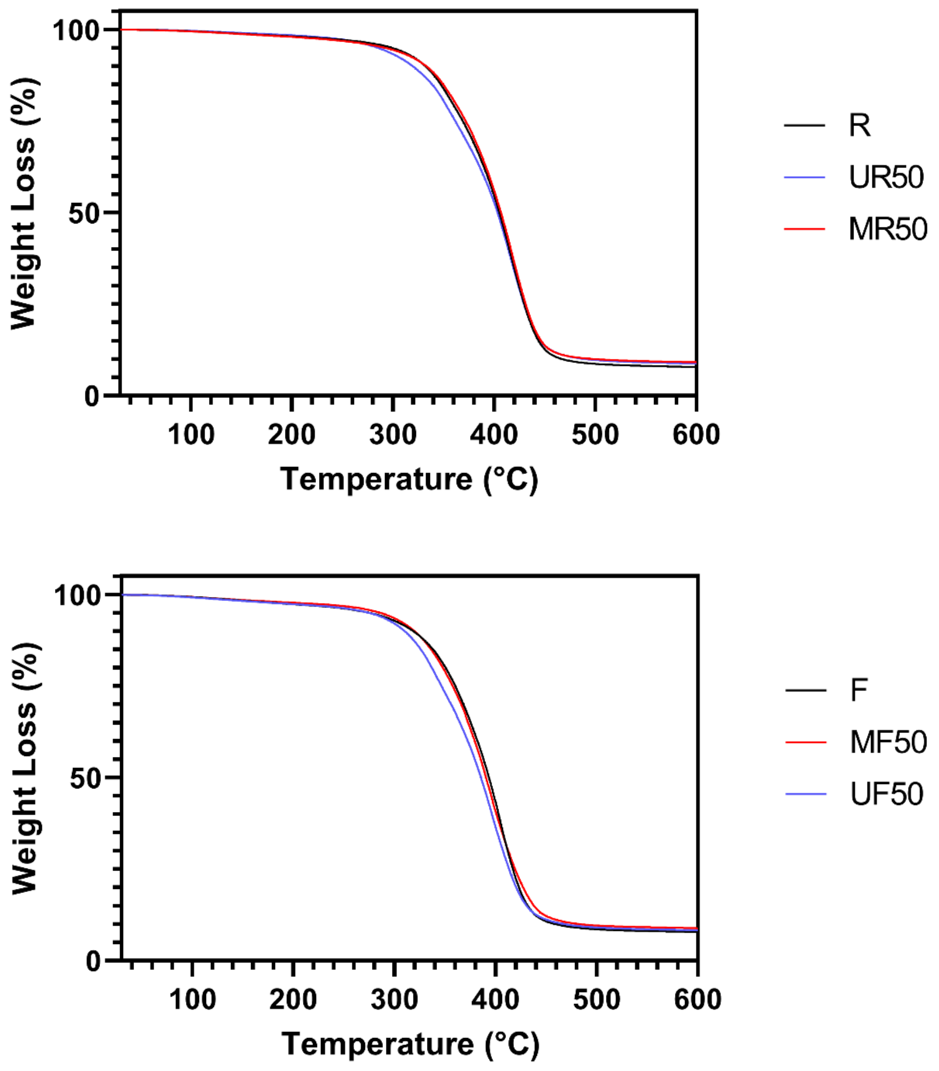

Figure S13. Thermogravimetric analysis of GO-loaded 3D printed resins.

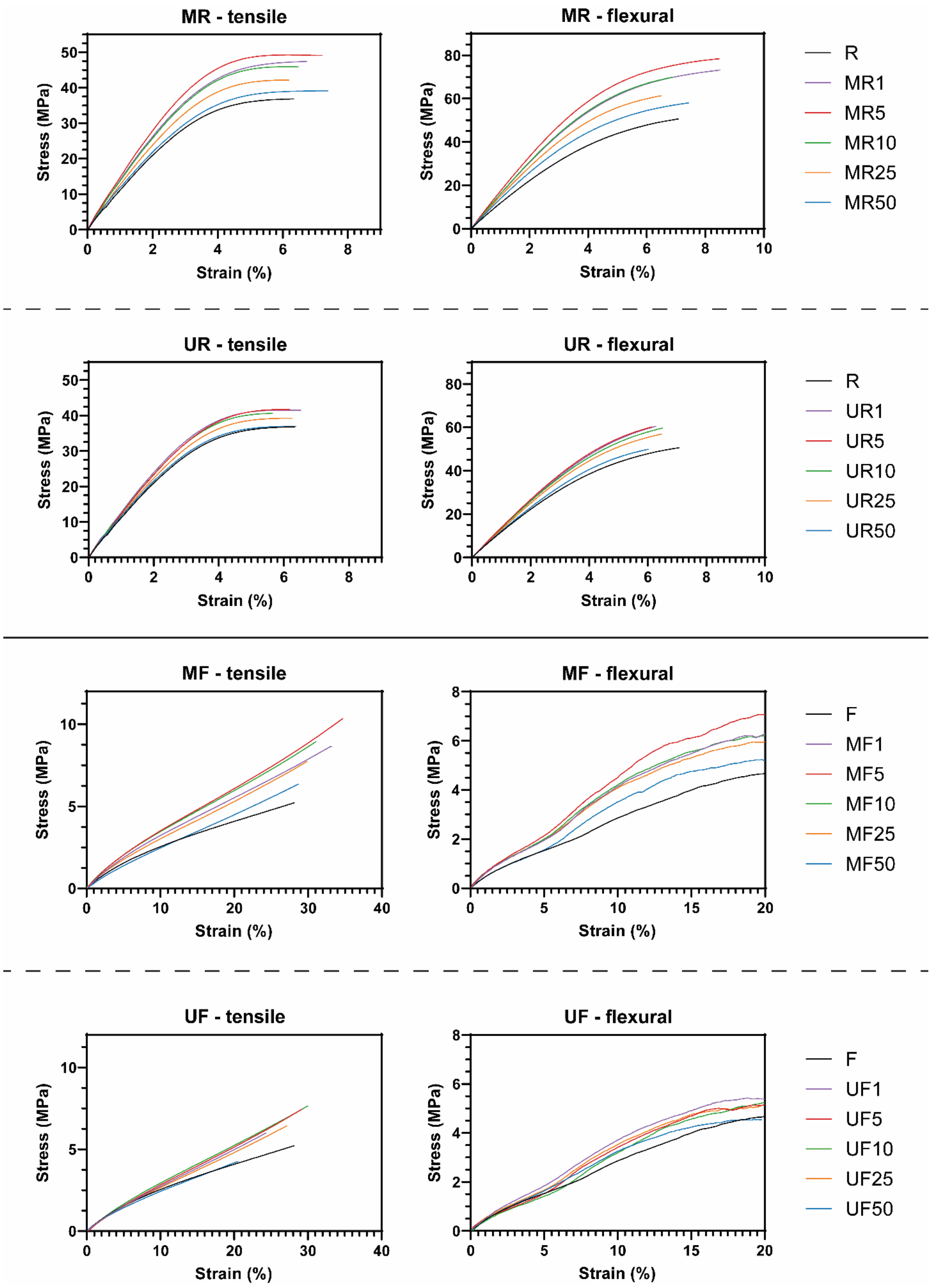

Figure S14. Tensile stress-strain curves. One representative sample has been plotted for each resin-filler system.

Sample	Tensile Testing				Flexural Testing			
	Elastic Modulus (GPa)	Elongation at break (%)	Tensile strength (MPa)	Tensile Toughness (J/mm^3)	Flexural Modulus (MPa)	Deformation at break (%)	Flexural strength (MPa)	Flexural Toughness (J/mm^3)
**R**	1.07 ± 0.03	5.9 ± 0.5	35.5 ± 0.8	1.46 ± 0.26	1.12 ± 0.06	7.1 ± 0.8	49.8 ± 2.6	2.21 ± 0.4
**MR50**	1.18 ± 0.02	6.5 ± 0.6	38.4 ± 0.8	1.67 ± 0.17	1.35 ± 0.02	6.1 ± 0.9	53.9 ± 4.0	2.06 ± 0.53
**MR25**	1.28 ± 0.05	5.9 ± 0.3	42.3 ± 0.7	1.79 ± 0.25	1.46 ± 0.04	6.3 ± 1.0	59.8 ± 3.8	2.40 ± 0.59
**MR10**	1.38 ± 0.02	6.5 ± 0.6	45.7 ± 1.7	2.13 ± 0.30	1.59 ± 0.06	6.8 ± 0.8	69.6 ± 3.4	3.00 ± 0.58
**MF5**	1.52 ± 0.02	6.8 ± 0.8	49.8 ± 0.8	2.20 ± 0.51	1.73 ± 0.03	7.6 ± 0.8	77.0 ± 2.1	3.84 ± 0.61
**MR1**	1.42 ± 0.01	7.0 ± 1.2	47.4 ± 1.2	2.39 ± 0.61	1.54 ± 0.05	8.5 ± 0.9	72.7 ± 3.1	4.11 ± 0.71
**UR50**	1.16 ± 0.02	5.8 ± 0.7	37.1 ± 0.8	1.28 ± 0.14	1.21 ± 0.03	6.0 ± 0.9	49.7 ± 2.3	1.83 ± 0.41
**UR25**	1.19 ± 0.02	5.3 ± 0.8	38.8 ± 0.3	1.33 ± 0.32	1.28 ± 0.04	6.1 ± 0.1	55.3 ± 3.2	2.05 ± 0.16
**UR10**	1.22 ± 0.02	5.4 ± 0.6	39.5 ± 1.0	1.42 ± 0.25	1.34 ± 0.02	5.6 ± 0.8	56.6 ± 3.5	1.89 ± 0.41
**UR5**	1.24 ± 0.05	6.1 ± 1.0	40.7 ± 1.2	1.73 ± 1.92	1.36 ± 0.02	5.5 ± 1.0	56.9 ± 6.5	2.16 ± 0.30
**UR1**	1.27 ± 0.03	6.5 ± 0.3	41.5 ± 0.9	1.92 ± 0.18	1.34 ± 0.02	6.6 ± 0.7	61.0 ± 2.1	2.53 ± 0.46

Table S3. Mechanical properties of rigid 3D printed GO nanocomposites. Data are expressed as mean ± SD (n = 5).

Sample	Tensile Testing				Flexural Testing		
	Elastic Modulus (MPa)	Elongation at break (%)	Tensile strength (MPa)	Tensile Toughness (MJ)	Flexural Modulus (MPa)	Deformation at break (%)	Flexural strength (MPa)
**F**	31.6 ± 1.5	25.4 ± 1.8	4.9 ± 0.3	0.73 ± 0.10	29.5 ± 1.7		5.0 ± 0.2
**MF50**	32.4 ± 2.3	28.2 ± 1.2	6.3 ± 0.3	0.94 ± 0.08	34.8 ± 2.2		5.2 ± 0.1
**MF25**	40.4 ± 3.4	28.8 ± 2.5	7.6 ± 0.6	1.18 ± 0.16	41.8 ± 2.0		6.0 ± 0.3
**MF10**	43.8 ± 3.6	31.9 ± 1.6	9.3 ± 0.5	1.58 ± 0.14	43.5 ± 1.1		6.1 ± 0.3
**MF5**	49.6 ± 3.3	32.5 ± 2.9	9.8 ± 0.9	1.71 ± 0.27	47.9 ± 2.9		7.0 ± 0.2
**MF1**	41.7 ± 0.5	31.4 ± 2.8	8.2 ± 0.7	1.41 ± 0.23	42.2 ± 2.5	No Break	6.2 ± 0.1
**UF50**	30.5 ± 1.8	21.5 ± 1.5	4.5 ± 0.4	0.53 ± 0.07	32.5 ± 0.9		4.6 ± 0.2
**UF25**	34.5 ± 2.6	26.2 ± 3.0	6.1 ± 0.7	0.87 ± 0.17	33.6 ± 2.7		5.0 ± 0.7
**UF10**	35.9 ± 2.6	28.1 ± 3.6	7.3 ± 0.9	1.11 ± 0.25	31.9 ± 1.8		5.3 ± 0.2
**UF5**	33.9 ± 1.5	27.4 ± 3.1	6.9 ± 0.9	1.01 ± 0.22	31.7 ± 2.1		5.2 ± 0.3
**UF1**	34.5 ± 1.3	26.7 ± 3.1	6.5 ± 0.8	0.94 ± 0.20	36.6 ± 1.7		5.2 ± 0.4

Table S4. Mechanical properties of flexible 3D printed GO nanocomposites. Data are expressed as mean ± SD (n = 5).

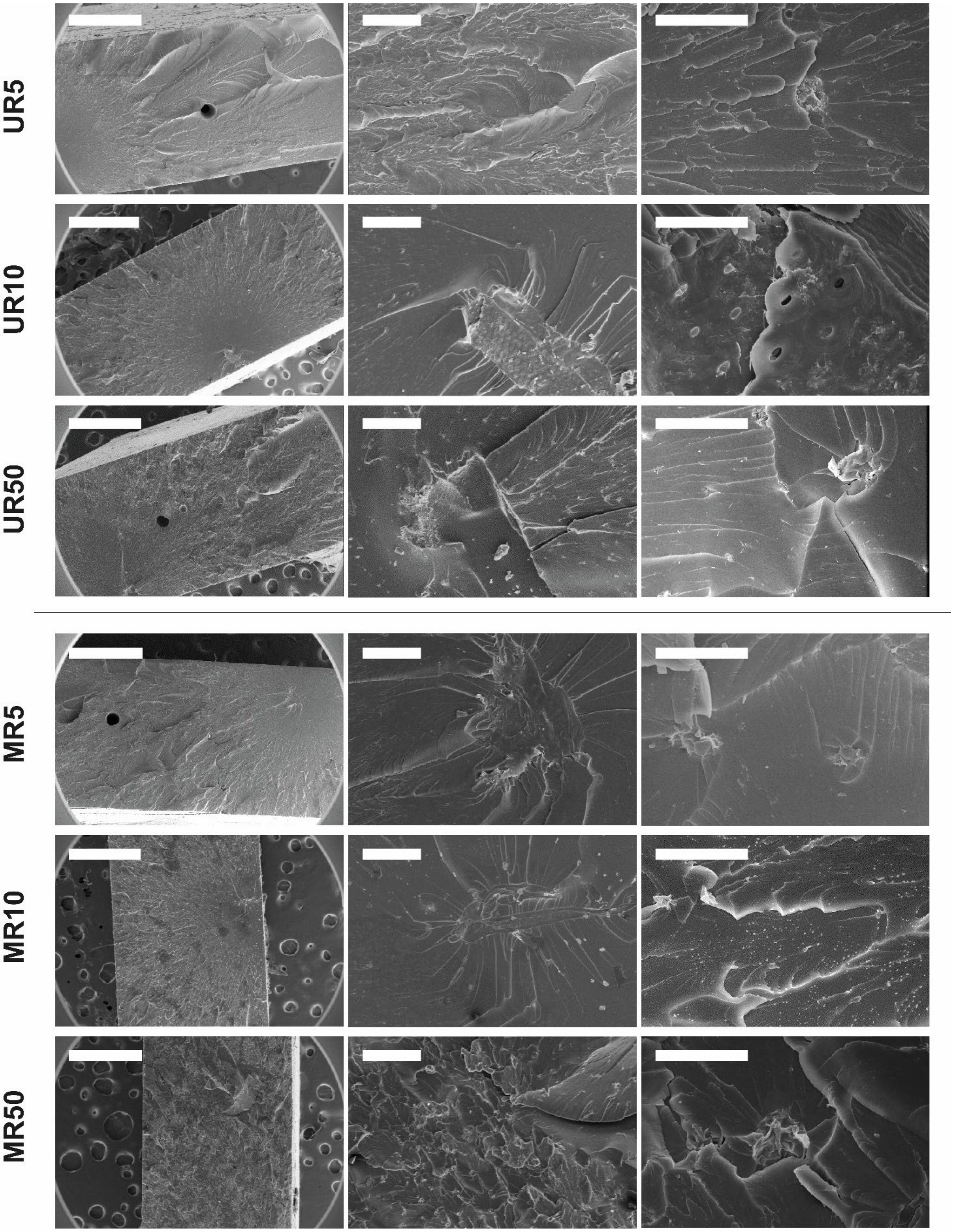

Figure S15. SEM images at the surface fracture of the composites made using resin R with 0.05, 0.1 and 0.5 wt.% GO (top panel) and 0.5, 1 and 5 wt.% GO@PBIA (bottom panel) ad different magnifications. Scale bars are 1 mm in the left column, 100 µm in the central column and 20 µm in the right column.

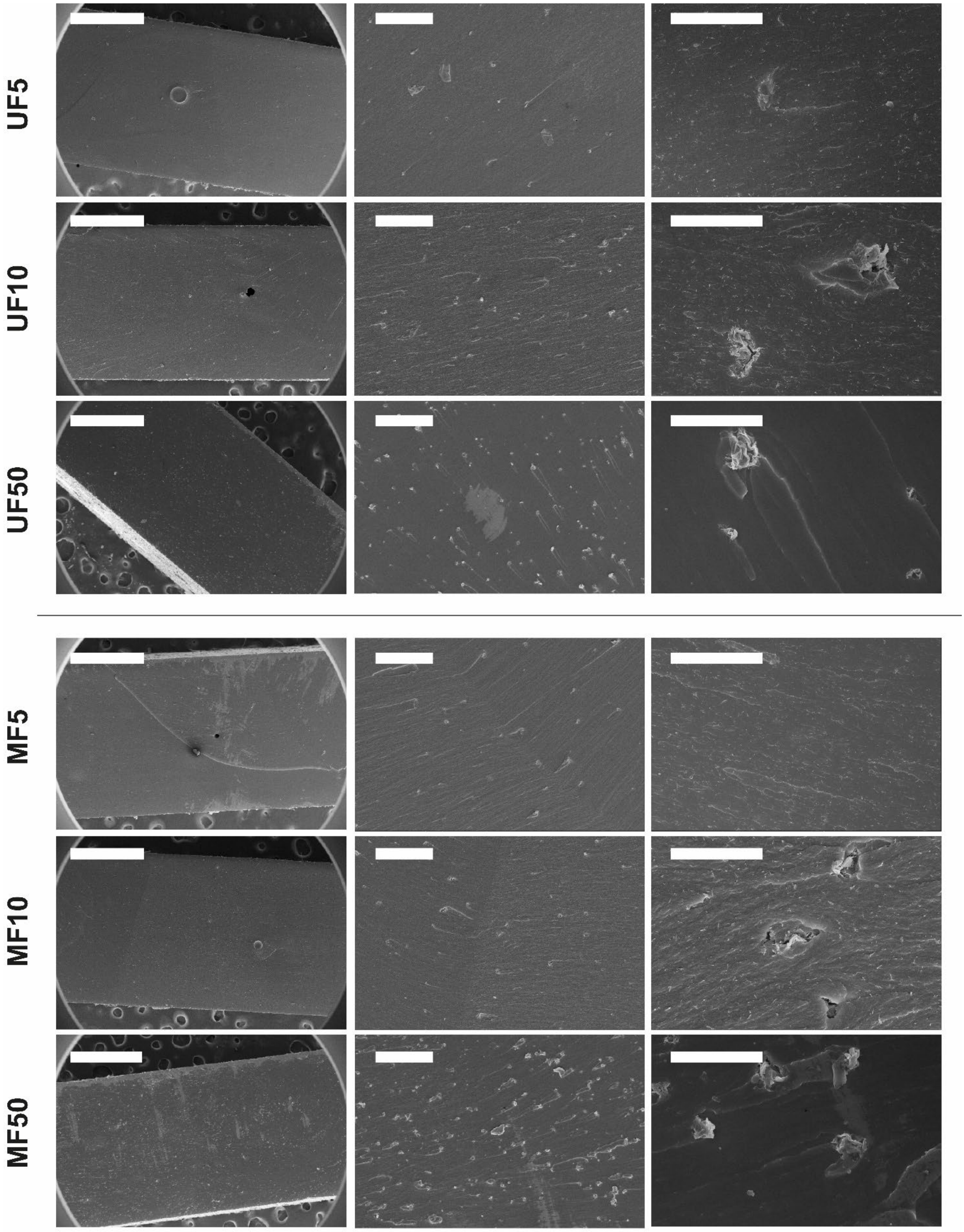

Figure S16. SEM images at the surface fracture of the composites made using resin F with 0.05, 0.1 and 0.5 wt.% GO (top panel) and 0.5, 1 and 5 wt.% GO@PBIA (bottom panel) ad different magnifications. Scale bars are 1 mm in the left column, 100 μm in the central column and 20 μm in the right column.
